# Accumulation of a Threonine Biosynthetic Intermediate Attenuates General Amino Acid Control by Accelerating Degradation of Gcn4 via Pho85 and Cdk8

**DOI:** 10.1371/journal.pgen.1004534

**Published:** 2014-07-31

**Authors:** Yashpal Rawal, Hongfang Qiu, Alan G. Hinnebusch

**Affiliations:** Laboratory of Gene Regulation and Development, Eunice Kennedy Shriver National Institute of Child Health and Human Development, National Institutes of Health, Bethesda, Maryland, United States of America; Rutgers Robert Wood Johnson Medical School, United States of America

## Abstract

Gcn4 is a master transcriptional regulator of amino acid and vitamin biosynthetic enzymes subject to the general amino acid control (GAAC), whose expression is upregulated in response to amino acid starvation in *Saccharomyces cerevisiae*. We found that accumulation of the threonine pathway intermediate β-aspartate semialdehyde (ASA), substrate of homoserine dehydrogenase (Hom6), attenuates the GAAC transcriptional response by accelerating degradation of Gcn4, already an exceedingly unstable protein, in cells starved for isoleucine and valine. The reduction in Gcn4 abundance on ASA accumulation requires Cdk8/Srb10 and Pho85, cyclin-dependent kinases (CDKs) known to mediate rapid turnover of Gcn4 by the proteasome via phosphorylation of the Gcn4 activation domain under nonstarvation conditions. Interestingly, rescue of Gcn4 abundance in *hom6* cells by elimination of *SRB10* is not accompanied by recovery of transcriptional activation, while equivalent rescue of UAS-bound Gcn4 in *hom6 pho85* cells restores greater than wild-type activation of Gcn4 target genes. These and other findings suggest that the two CDKs target different populations of Gcn4 on ASA accumulation, with Srb10 clearing mostly inactive Gcn4 molecules at the promoter that are enriched for sumoylation of the activation domain, and Pho85 clearing molecules unbound to the UAS that include both fully functional and inactive Gcn4 species.

## Author Summary

Transcriptional activator Gcn4 maintains amino acid homeostasis in budding yeast by inducing multiple amino acid biosynthetic pathways in response to starvation for any amino acid—the general amino acid control. Gcn4 abundance is tightly regulated by the interplay between an intricate translational control mechanism, which induces Gcn4 synthesis in starved cells, and a pathway of phosphorylation and ubiquitylation that mediates its rapid degradation by the proteasome. Here, we discovered that accumulation of a threonine biosynthetic pathway intermediate, β-aspartate semialdehyde (ASA), in *hom6Δ* mutant cells impairs general amino acid control in cells starved for isoleucine and valine by accelerating the already rapid degradation of Gcn4, in a manner requiring its phosphorylation by cyclin-dependent kinases Cdk8/Srb10 and Pho85. Interestingly, our results unveil a division of labor between these two kinases wherein Srb10 primarily targets inactive Gcn4 molecules—presumably damaged under conditions of ASA excess—while Pho85 clears a greater proportion of functional Gcn4 species from the cell. The ability of ASA to inhibit transcriptional induction of threonine pathway enzymes by Gcn4, dampening ASA accumulation and its toxic effects on cell physiology, should be adaptive in the wild when yeast encounters natural antibiotics that target Hom6 enzymatic activity.

## Introduction

Cells undergo rapid transcriptional reprogramming in response to environmental changes by mobilizing transcriptional activators and repressors. Transcriptional activators function by binding to specific DNA sequences (UAS elements in yeast) and recruiting transcriptional cofactor proteins/complexes that remove repressive chromatin structure and directly recruit the transcriptional machinery to the promoters of genes under their control. Various mechanisms have been elucidated for stimulating activator function in response to environmental signals, including dissociation from a repressor, as in the case of yeast Gal4 [1], or increased entry into the nucleus as described for Pho4 and Gln3 [2]. The yeast activator Gcn4 is regulated by a unique translational control mechanism that rapidly increases the rate of Gcn4 synthesis in response to limitation for any amino acid—the conditions where increased transcription of amino acid biosynthetic genes under Gcn4 control is essential to maintaining cell growth. Gcn4 is also negatively regulated by a pathway that evokes its phosphorylation, ubiquitylation, and degradation by the proteasome, such that continued high-level translation of *GCN4* mRNA is required to sustain induction of Gcn4 protein and its target genes. Together, these systems provide for reversible, short-lived induction of Gcn4, except under conditions of extreme starvation—in which protein synthesis is strongly impaired—where Gcn4 turnover is attenuated (reviewed in [3]). In addition to stimulating the transcription of genes encoding enzymes representing all of the amino acid biosynthetic pathways—the regulatory response dubbed general amino acid control (GAAC)— one-tenth or more of the yeast genome is induced by Gcn4, including genes involved in producing amino acid precursors, mitochondrial carrier proteins, vitamins and cofactors, amino acid transporters, autophagy, or the metabolism of purine, glycogen, and trehalose [4], [5].

The induction of Gcn4 expression at the translational level in amino acid-starved cells requires the protein kinase Gcn2, which is activated by uncharged tRNAs cognate to the limiting amino acid. Gcn2's sole substrate in yeast is the α subunit of general translation initiation factor 2 (eIF2). In its GTP-bound form, eIF2 delivers charged methionyl initiator tRNA (Met-tRNA_i_^Met^) to the small (40S) ribosomal subunit in the first step of translation initiation. The inactive eIF2-GDP complex is released at the end of the process and must be recycled to eIF2-GTP by the guanine nucleotide exchange factor eIF2B. Phosphorylation of eIF2α on serine-51 by Gcn2 converts eIF2-GDP from substrate to inhibitor of eIF2B, impeding the formation of the eIF2-GTP-Met-tRNA_i_^Met^ ternary complex (TC). While this reduces the rate of bulk protein synthesis and limits amino acid consumption, it specifically induces translation of *GCN4* mRNA owing to specialized regulatory sequences (upstream ORFs) present in the mRNA leader that couple reduced TC concentration to increased initiation at the *GCN4* AUG start codon [3]. The newly synthesized Gcn4 enters the nucleus—a constitutive process for this activator [6], binds to the UAS elements of its target genes and recruits multiple cofactors to the promoter. The recruited cofactors include the nucleosome remodeling complexes SWI/SNF and RSC; and the SAGA and Mediator complexes, which carry out histone acetylation and/or function as adaptors to recruit general transcription factors and RNA polymerase II (PolII), culminating in increased assembly of transcription initiation complexes and elevated transcription of the coding sequences (CDS) [7]–[10].

In nutrient-replete yeast cells, and under conditions of moderate amino acid limitation, where Gcn2 is activated and translation of *GCN4* mRNA induced, Gcn4 is a highly unstable protein owing to its ubiquitylation by ubiquitin ligase SCF^CDC4^ and attendant degradation by the proteasome [11]–[13]. This process helps to maintain Gcn4 at a low, basal level in nonstarved cells, and allows rapid restoration of the basal level when the translation rate of *GCN4* mRNA is repressed by replenishing amino acids in starved cells. Rapid degradation of Gcn4 in sated or moderately starved cells requires its phosphorylation by the CDKs Cdk8/Srb10 and Pho85, with Pho85 making the greater contribution [12], [14]. In severely starved cells, Pho85's contribution to Gcn4 turnover is essentially eliminated, owing to the destabilization and consequent disappearance of its cyclin Pcl5 [15], [16], which accounts in large part for the stabilization of Gcn4 under these conditions. By contrast, Srb10 contributes to Gcn4 turnover under all conditions examined, making the minor contribution in sated or moderately starved cells but the major contribution in severely starved cells (where Pho85 is inactive) [14].

Despite its lesser importance in Gcn4 turnover, Srb10 appears to be responsible for clearing the fraction of Gcn4 that is sumoylated on Lys residues 50 and 58. It appears that sumoylation of Gcn4 on K50/K58 reduces its occupancy at the UAS elements of target genes in the early stages of GAAC induction during moderate starvation for Ile/Val imposed with the inhibitor sulfometuron (SM). However, the higher levels of UAS-bound unsumoylated Gcn4 that result from Arg substitutions of K50/K58 do not evoke increased PolII occupancy or higher transcription rates under these conditions in otherwise WT cells [17]. As sumoylation of transcription factors can inhibit transcriptional activation by impairing their ability to recruit RNA polymerase [18], sumoylation of Gcn4 might impair its activator function to dampen the general control response.

There is evidence that phosphorylation of Gcn4 by Srb10 or Pho85 reduces its activation function and that the phosphorylated species must be ubiquitylated and degraded by the proteasome to maintain WT basal expression of Gcn4 target genes under nonstarvation conditions. Thus, blocking proteasomal degradation of Gcn4 reduces target gene transcription in non-starved cells in a manner suppressed by eliminating CDK phosphorylation sites in Gcn4 or deleting both Srb10 and Pho85 [19]. It is unclear whether the accumulation of phosphorylated Gcn4 also impairs transcriptional activation under inducing conditions of amino acid starvation. The phosphorylation of Gcn4 by both kinases appears to be nucleus-localized, as Gcn4 mutants lacking nuclear localization signals are stabilized [6]. Srb10 is associated with the Mediator coactivator complex [20], which phosphorylates the CTD of the largest subunit of RNA polymerase II, Rpb1 [21]. The fact that Gcn4 recruits Mediator to the promoter [7], [8] is consistent with the possibility that Gcn4 participates in down-regulating its own function and stability by recruiting at least one of its inactivating CDKs [14].

**Figure 1:**
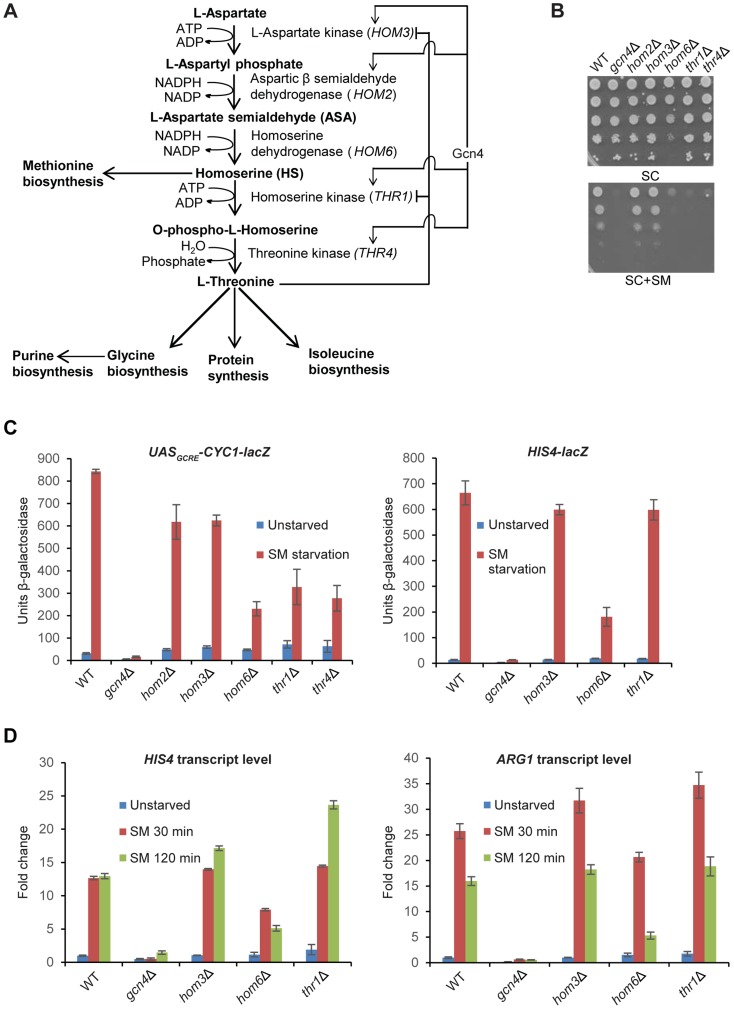
*hom6Δ* impairs GAAC in cells starved for Ile/Val. (**A**) Schematic diagram of the threonine biosynthesis pathway in *Saccharomyces cerevisiae*. Feedback inhibition by threonine of aspartate kinase (*HOM3*) and homoserine kinase (*THR1*) and transcriptional induction of certain pathway genes by Gcn4 under the GAAC are shown. (**B**) SM-sensitivity of *hom6Δ*, *thr1Δ*, and *thr4Δ* mutants. Parental WT strain BY4741/F729, *gcn4Δ* mutant F731, and deletion mutants lacking the indicated threonine biosynthetic gene (F2057, F1929, F941, F2056 and F926) were cultured overnight in SC-Ile/Val containing 2.5 mM threonine, washed and resuspended in sterile water at A_600_ = 1.0, and 10-fold serial dilations were spotted on agar plates of the same growth medium (SC) or medium also containing 0.5 µg/ml sulfomeutron methyl (SC+SM) and incubated at 30°C for 2d. (**C**) Yeast strains from (B) were transformed with pHYC2 (*UAS_GCRE_-CYC1-lacZ*) or p367 (*HIS4-lacZ*) and cultured overnight in SC-Ura/Ile/Val containing 2.5 mM threonine. Duplicate cultures were diluted at A_600_ = 0.5 in SC-Ura/Ile/Val medium containing 1 mM threonine, and one set was harvested after 6 h at 30°C (Unstarved). For the duplicate set, SM was added to 0.5 µg/mL after 2.5 h and incubation continued another 6 h before harvesting. β-galactosidase activity (nmole of ONPG cleaved per min per mg) was measured in WCEs for three independent transformants of each strain, and mean and S.E.M. values are plotted. (**D**) WT (BY4741), *gcn4Δ* (F731), *hom3Δ* (F1929), *hom6Δ* (F941) and *thr1Δ* (F2056) strains were cultured as in (C) except that cultures were harvested at A_600_ = 0.4–0.6 after doubling at least twice (Unstarved) or treated with SM and cultured an additional 30 min or 120 min. Total RNA was purified and used for cDNA synthesis and using the appropriate fluorescently-labeled Taqman probes *HIS4, ARG1*, and *ACT1* mRNAs were quantified by real-time qPCR. The levels of *HIS4* or *ARG1* mRNAs were normalized to those of *ACT1* mRNA and expressed relative to the value determined for WT unstarved cells. Mean and S.D. values determined from two independent cultures are plotted.

Biosynthesis of threonine in yeast, as in other microorganisms and several plants, is a five-step pathway initiated with L-aspartic acid as the primary substrate [22],[23] (Fig. 1A). The absence of the threonine biosynthetic pathway in humans makes it a valuable target for drug development against fungal pathogens [24]. Transcription of at least 4 genes of the threonine pathway, *HOM3*, *HOM2, THR1* and *THR4* is under Gcn4 control (Fig. 1A) [4], [25]. Threonine biosynthesis is also subject to feedback inhibition by threonine, which inhibits the activity of the first enzyme in the pathway, aspartate kinase (Hom3), and also partially inhibits homoserine kinase (Thr1) (Fig. 1A) [26]. Peptide prolyl isomerase FKBP12 participates in the feedback inhibition of Hom3 by physical interaction between these two proteins [27], [28].

Besides threonine auxotrophy, *thr1Δ* and *thr4Δ* mutants exhibit myriad phenotypes that result from accumulation of the pathway intermediate homoserine (HS), as they are mitigated in *thr1Δ hom3Δ* double mutants that cannot produce HS (Fig. 1A) [29]. Accumulation of the substrate of Hom6, β-aspartate semialdehyde (ASA), also is toxic, as releasing feedback inhibition of Hom3 is lethal in *hom6Δ* cells (that accumulate ASA) in a manner rescued by simultaneously blocking ASA synthesis by eliminating Hom2 or Hom3 [27], [29]. However, *hom6Δ* mutants do not share all phenotypes of *thr1Δ* and *thr4Δ* mutants, and *hom6Δ* suppresses those unique to the latter mutants, implicating HS and ruling out a role for ASA in conferring many defects displayed by *thr1Δ* and *thr4Δ* cells. There is circumstantial evidence that HS toxicity results from its incorporation into proteins in place of threonine, which might evoke increased degradation of the HS-substituted proteins by the proteasome [29].

Previously, we screened the entire library of viable haploid deletion mutants of *Saccharomyces cerevisiae* for sensitivity to SM (SM^S^ phenotype) to identify genes required for a robust GAAC, which allowed us to implicate various cofactors in the mechanism of transcriptional activation by Gcn4 [7], [30], and certain vacuolar sorting proteins (Vps) in maintaining high-level Gcn4 activation function in cells starved for Ile/Val [13]. In the course of that work, we also discovered that *hom6Δ, thr1Δ*, and *thr4Δ* mutants are also SM^S^, and undertook here to elucidate the mechanisms underlying this phenotype. In fact, it had been shown previously that *thr1Δ* mutants are SM^S^, and that this phenotype is suppressed by deleting *HOM3*. As SM evokes derepression of threonine pathway enzymes by Gcn4 [4] (Fig. 1A), the SM^S^ phenotype of *thr1Δ* mutants was attributed to Hom3-dependent accumulation of HS, and its attendant toxicity to cellular processes, when *HOM3* and *HOM2* transcription is induced by Gcn4 [29]. This explanation would not apply to *hom6Δ* cells, however, which cannot produce HS, leading us to examine whether the SM^S^ phenotype in this instance results from ASA accumulation and impairment of the GAAC response. The results of our analysis indicate that ASA accumulation indeed attenuates GAAC, by accelerating further the already rapid degradation of Gcn4 triggered by the CDKs Pho85 and Srb10. They further suggest that Srb10 functions primarily in efficient clearance of inactive Gcn4 molecules, enriched for sumoylated species, whereas Pho85 clears unsumoylated, highly functional Gcn4 in addition to defective species.

## Results

### *hom6Δ* cells are defective in transcriptional activation by Gcn4

As noted above and displayed in Fig. 1B, yeast deletion mutants lacking *HOM6, THR1*, or *THR4* are sensitive to sulfometuron methyl (SM), which evokes starvation for isoleucine and valine (Ile/Val) by inhibition of the *ILV2-*encoded biosynthetic enzyme [4]. At the SM concentration employed, growth of the *hom6Δ, thr1Δ* and *thr4Δ* strains is impaired to an extent similar to that of the *gcn4Δ* strain, lacking the activator of GAAC. Unlike these mutants, the *hom3Δ* and *hom2Δ* mutants grow like the wild-type (WT) strain on SM-containing medium (Fig. 1B, SC+SM). These findings indicate that *thr1Δ, thr4Δ*, and *hom6Δ* strains, but not *hom3Δ* and *hom2Δ* mutants, are sensitive to Ile/Val starvation imposed by SM. Moreover the *hom6Δ* mutant grows more slowly than WT (Slg^-^ phenotype) even on medium lacking SM (Fig. 1B, SC).

To determine whether the SM^S^ phenotypes of the *thr1Δ, thr4Δ*, and *hom6Δ* mutants reflect defective transcriptional activation by Gcn4, we measured induction of a *UAS_GCRE_-CYC1-lacZ* reporter, driven by the *CYC1* promoter and tandem Gcn4 binding sites from *HIS4* (the *UAS_GCRE_*) replacing the endogenous *CYC1* UAS; and of a *HIS4-lacZ* reporter containing the native *HIS4* 5′-noncoding region. (*HIS4* is a known Gcn4 target gene [5], [31].) As expected, treatment with SM for 6 h evokes a strong increase in *UAS_GCRE_-CYC1-lacZ* reporter expression in WT, but not in *gcn4Δ* cells (Fig. 1C). Smaller induction ratios were observed for all five mutants of the threonine pathway, with the largest defect seen for the *hom6Δ* strain (∼75% reduction of induced *UAS_GCRE_-CYC1-lacZ* expression) and the smallest defects observed for the *hom3Δ* and *hom2Δ* mutants (∼25% reductions) (Fig. 1C, left). In the case of the *HIS4-lacZ* reporter, the *hom6Δ* mutant, but not the *hom3Δ* or *thr1Δ* strains, displayed a marked (∼75%) reduction in induction by SM (Fig. 1C, right).

To confirm these findings, we measured induction of native mRNAs for *HIS4* and *ARG1* (another known Gcn4 target gene). Consistent with the *HIS4-lacZ* data, we observed induction defects for the *hom6Δ* mutant, but not the *hom3Δ* or *thr1Δ* strains, for both mRNAs (Fig. 1D). The magnitude of the induction defect in the *hom6Δ* mutant was considerably greater after 120 min versus 30 min of SM treatment, displaying ∼60% and ∼67% reductions for *HIS4* and *ARG1* mRNAs, respectively, at the longer incubation time, even though full induction of both mRNAs was achieved by 30 min of SM treatment in WT cells (Fig. 1D).

The foregoing results indicate that the SM-sensitivity of the *hom6Δ* mutant reflects a substantial defect in GAAC resulting from reduced transcriptional activation by Gcn4, which becomes more severe as starvation proceeds. By contrast, the other four threonine pathway mutants exhibit smaller defects in transcriptional activation, and the *hom3Δ* and *thr1Δ* strains actually display no detectable impairment of *HIS4* and *ARG1* induction by SM. The strong SM^S^ phenotypes of the *thr1Δ* and *thr4Δ* mutants (Fig. 1B) can be reconciled with their moderate GAAC defects (Figs. 1C–D) by recalling that they accumulate the toxic intermediate HS, and that Gcn4-mediated induction of *HOM2* and *HOM3* under SM-induced starvation conditions is expected to elevate HS production in these strains (Fig. 1A), in the manner proposed previously for *thr1Δ* cells [29]. By contrast, induction of the threonine pathway during SM treatment in *hom3Δ* or *hom2Δ* mutants should have no effect on cell growth (as observed in Fig. 1B) because they cannot produce HS.

### The catalytic activity of Hom6 is required for a robust GAAC response

**Figure 2:**
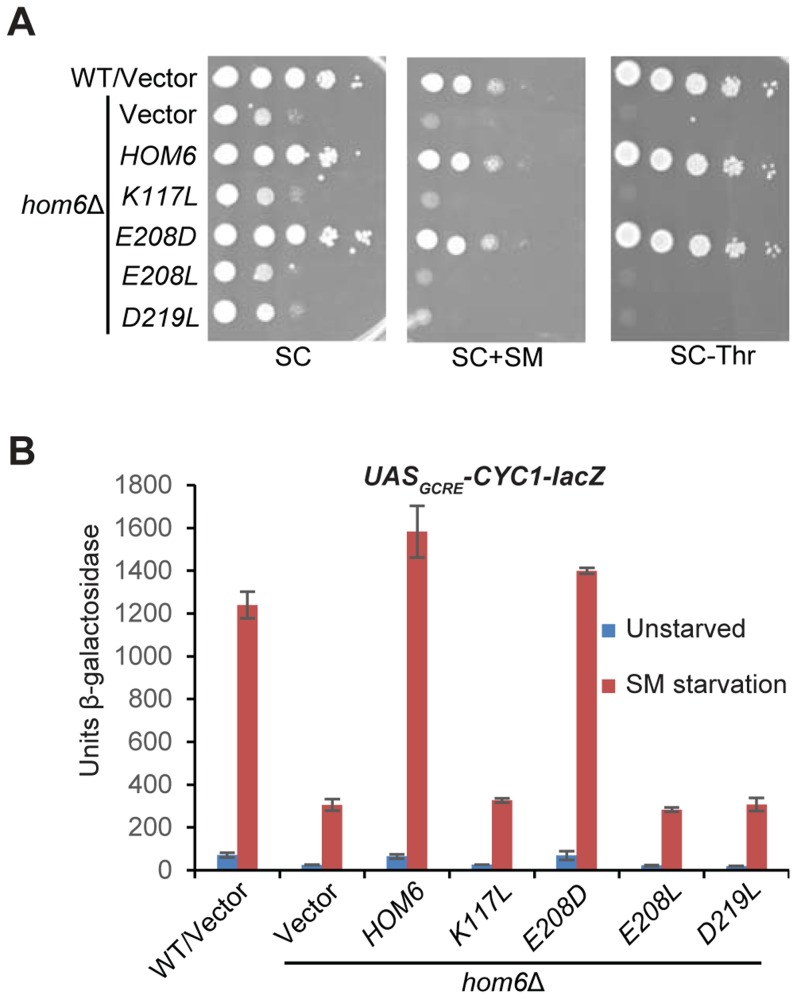
Hom6 catalytic activity is required for robust GAAC in Ile/Val-starved cells. (**A**) The growth of WT (BY4741) and *hom6Δ* (F941) strains transformed with vector (YCplac111) and F941 (*hom6Δ*) transformed with single-copy plasmids carrying WT *HOM6* (pYPR010) or active-site *hom6* alleles *K117L* (pYPR018), *E208D* (pYPR020), *E208L* (pYPR022), or *D219L* (pYPR024) was analyzed essentially as in Fig. 1B except that growth on SC-Leu/Thr medium (SC-Thr) was also examined. (**B**) Transformants of the strains in (A) harboring pHYC2 were analyzed for β-galactosidase activity as in Fig. 1C. Means and S.E.Ms were calculated from three independent transformants of each strain.

The *HOM6* product, homoserine dehydrogenase (HSD), converts β-aspartate semialdehyde (ASA) into HS. If the GAAC defect in *hom6Δ* cells results from the absence of this reaction, then *hom6* mutants that produce catalytically defective HSD should display a strong GAAC defect. Based on a crystal structure of yeast HSD, 4 active site substitutions were generated that were previously characterized for their effects on HSD catalytic activity in vitro [32]. We introduced the corresponding mutations into plasmid-borne *HOM6* and examined the ability of the mutant alleles to complement the transcriptional activation defects of *hom6Δ* cells. As expected, introduction of WT *HOM6* complemented the Slg^-^ and SM^S^ phenotypes on media containing threonine, and the failure to grow on medium lacking threonine, of the *hom6Δ* strain (Fig. 2A, SC, SC+SM and SC-Thr, respectively). Except for the *E208D* allele, the plasmid-borne *hom6* alleles encoding HSD active site substitutions abolished complementation of the threonine auxotrophy and SM-sensitivity of the *hom6Δ* strain (Fig. 2A). Consistent with this, the three defective alleles failed to restore SM-induction of the *UAS_GCRE_-CYC1-lacZ* reporter, whereas *E208D* restored a WT level of induction (Fig. 2B). Interestingly, the previously determined kinetic parameters of the *hom6-E208D* product indicated a reduced substrate affinity, but high-level catalytic activity, in comparison to WT HSD [32]. Accordingly, our results demonstrate that HSD catalytic activity is required for a robust GAAC response. We presume that the diminished substrate affinity of the *hom6-E208D* mutant does not significantly reduce the rate of converting ASA to HS in living cells.

### Accumulation of β-aspartate semialdehyde in *hom6Δ* cells impairs GAAC

**Figure 3:**
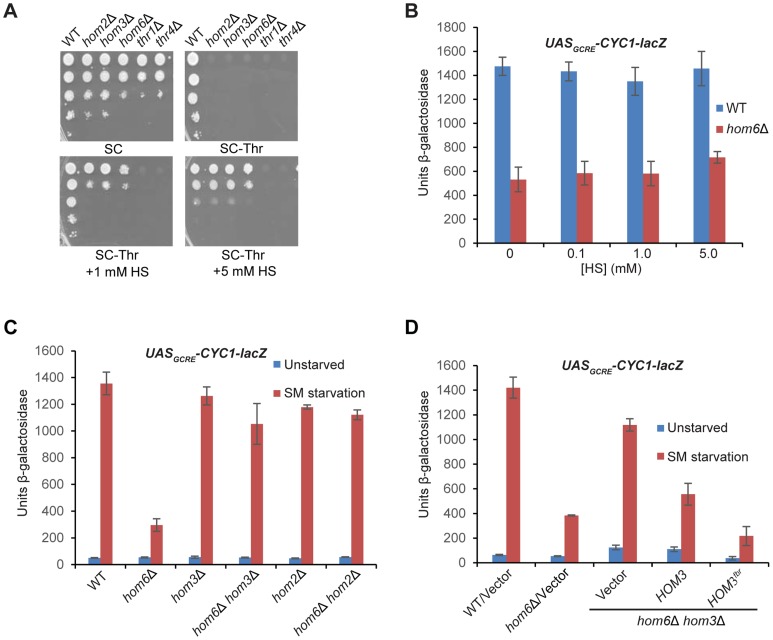
Accumulation of aspartate semialdehyde in *hom6Δ* cells impairs GAAC. (**A**) Yeast strains described in Fig. 1B were analyzed for growth in spotting assays using SC medium containing Ile/Val and 1 mM Thr (SC), the same medium lacking Thr (SC-Thr) either with or without addition of homoserine (HS) at the indicated concentrations. (**B–D**) Transformants of the indicated relevant genotypes harboring pHYC2 were analyzed for *UAS_GCRE_-CYC1-lacZ* expression as in Fig. 1C except for the addition of HS and the analysis of only SM-treated cultures in (B). pHYC2 transformants of the following strains were examined: (B) WT (BY4741) and *hom6Δ* (F941); (C) WT (BY4741), *hom6Δ* (YR001), *hom3Δ* (F1929), *hom2Δ* (F2057), *hom6Δ hom2Δ* (YR022), and *hom6Δ hom3Δ* (YR003); (D) WT (BY4741) and *hom6Δ* (YR001) strains transformed with vector pRS313, and *hom6Δ hom3Δ* strain YR003 transformed with pRS313, low copy (lc) *HOM3* plasmid pYPR028, or lc *HOM3^fbr^* plasmid pYPR030. Means and S.E.Ms were calculated from three independent transformants of each strain.

We asked next whether the requirement for HSD activity for the GAAC response reflects a requirement for HS synthesis or, rather, the need to prevent accumulation of ASA. If the inability to produce HS is the salient defect, then supplementing *hom6Δ* cells with HS should restore their GAAC response. We found that a supplement of 1 mM HS restores growth on SC-Thr medium for the *hom3Δ, hom2Δ* and *hom6Δ* strains, but not for the *thr1Δ* or *thr4Δ* strains (Fig. 3A), consistent with the position of Thr1 and Thr4 downstream of HS production in the Thr pathway (Fig. 1A). A supplement of 5 mM HS was required to confer growth of the *hom3Δ, hom2Δ* and *hom6Δ* strains indistinguishable from that of WT; although this elevated HS concentration retards the growth of WT cells (Fig. 3A), presumably reflecting HS toxicity [29]. Importantly, HS supplementation did not rescue the defective SM-induction of the *UAS_GCRE_-CYC1-lacZ* reporter in the *hom6Δ* mutant (Fig. 3B), indicating that its GAAC defect does not result from the inability to produce HS.

If accumulation of the Hom6/HSD substrate (ASA) in *hom6Δ* cells is responsible for the GAAC defect, then the GAAC response should be restored by preventing ASA production by eliminating Hom2 or Hom3; moreover, the GAAC defect should be exacerbated by eliminating feedback inhibition of the Hom3 product (Fig. 1A). Indeed, deleting *HOM2* or *HOM3* in the *hom6Δ* mutant restored SM-induction of the *UAS_GCRE_-CYC1-lacZ* reporter essentially to the same levels observed in the *hom2Δ* or *hom3Δ* single mutants (Fig. 3C). Introducing WT *HOM3* into the *hom3Δ hom6Δ* strain reinstated a defect in SM-induction of *UAS_GCRE_-CYC1-lacZ* similar to that seen in the *hom6Δ* single mutant (Fig. 3D). Importantly, a relatively greater induction defect was observed when the feedback-resistant allele *hom3-E282D* (dubbed *HOM3^fbr^*) was introduced instead into the *hom3Δ hom6Δ* strain (Fig. 3D, cf. last two columns). As expected, introduction of *HOM3^fbr^* into the *hom3Δ hom6Δ* strain confers a strong Slg^-^ phenotype (Fig. S1), owing to accumulation of ASA and its toxic effects on cell growth [27]. These findings demonstrate that the GAAC defect in *hom6Δ* cells results from ASA accumulation.

### ASA accumulation in *hom6Δ* cells reduces Gcn4 abundance

**Figure 4:**
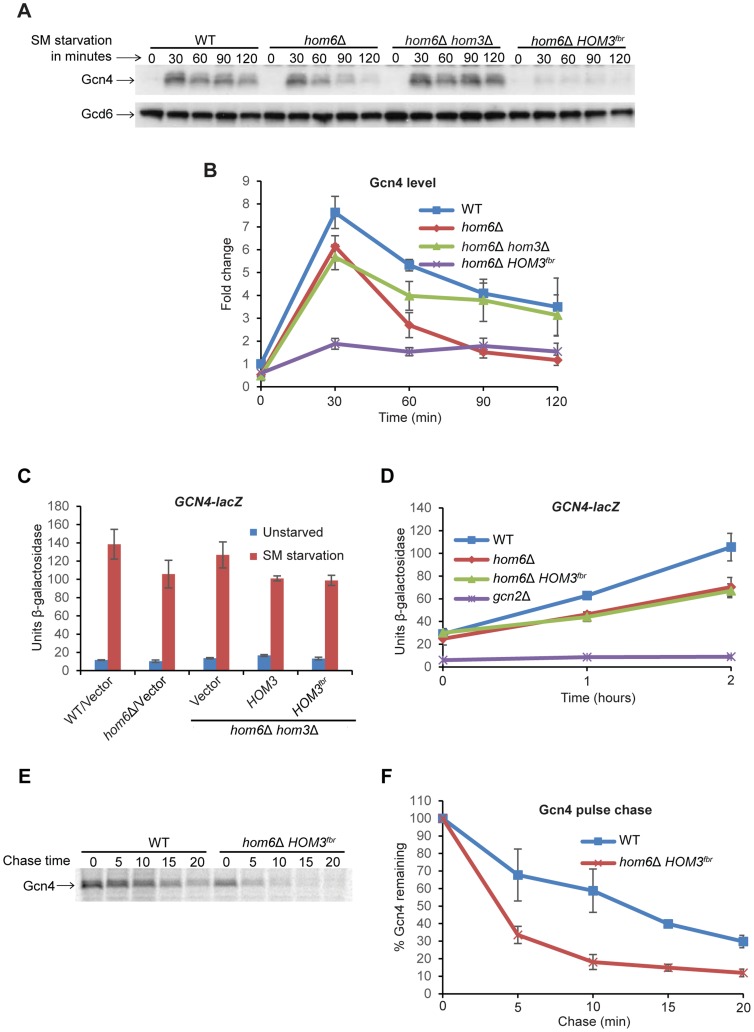
Accumulation of ASA lowers Gcn4 abundance and stability. (**A**) WT (BY4741) and *hom6Δ* (YR001) strains transformed with vector (pRS313) and *hom6Δ hom3Δ* strain YR003 transformed with vector or lc *HOM3^fbr^* plasmid pYPR030 (indicated as *hom6Δ hom3Δ* and *hom6Δ HOM3^fbr^* respectively) were cultured in SC-His/Ile/Val for at least two doublings to A_600_ = 0.4–0.6 and subjected to SM treatment (0.5 µg/ml) for the indicated times. WCEs were extracted under denaturing conditions and subjected to Western analysis with α-Gcn4 and α-Gcd6 antibodies. (**B**) Western signals from (A) were quantified, Gcn4 signals were normalized to Gcd6 signals, and the Gcn4/Gcd6 ratios were expressed relative to that measured for the WT strain without SM treatment. Means and S.E.Ms were calculated from three independent transformants of each strain. (**C**) Yeast strains described in Fig. 3D transformed with p180 were analyzed for *GCN4-lacZ* expression as in Fig. 1C. Means and S.E.Ms were calculated from three independent transformants of each strain. (**D**) p180 transformants of strains from (C) and *gcn2Δ* strain H2931 were analyzed for *GCN4-lacZ* expression after SM treatment for the indicated times. Means and S.E.Ms were calculated from three independent transformants of each strain. (**E**) The WT and *hom6Δ HOM3^fbr^* strains from (A) were cultured in SC-His/Ile/Val, collected and resuspended in SC-His/Ile/Val/Met containing SM (1 µg/ml) for 15 min, and labeled with [^35^S] methionine/cysteine for 15 min. Cells were collected and resuspended in SC-His/Ile/Val containing Met and Cys (both at 10 mM) and aliquots were removed after the indicated times of chase. Aliquots of WCEs containing equal cpm were immunoprecipitated with α-Gcn4 antibodies, immunocomplexes were collected with protein A-agarose beads and resolved by SDS-PAGE, and the [^35^S]-Gcn4 signals were quantified by phosphorimaging. (**F**) Gcn4 signals from (E) are plotted relative to the value at 0 min of chase (100%). Means and S.D.s were calculated from two independent experiments.

We sought next to determine whether ASA accumulation impairs the GAAC by reducing Gcn4 abundance. Starvation for Ile/Val by SM rapidly increases Gcn4 synthesis by inducing the translation of *GCN4* mRNA [3]. Western analysis of WT cells reveals the expected rapid induction of Gcn4 after only 30 min of SM treatment, with a gradual decline in abundance as starvation continues up to 120 min [9] (Fig. 4A and B). Gcn4 abundance was decidedly reduced over much of the time course of SM treatment in vector transformants of the *hom6Δ* strain, again reaching its lowest level at 120 min of SM treatment. This reduction in Gcn4 abundance was mitigated by the absence of *HOM3* in the *hom6Δ hom3Δ* double mutant, and exacerbated in transformants of the double mutant harboring feedback-resistant *HOM3^fbr^*, in which ASA accumulation is eliminated or exacerbated, respectively (Fig. 4A and B). Even after only 30 min of SM treatment, the *hom6Δ HOM3^fbr^* strain displayed low-level Gcn4 similar to that observed in *hom6Δ*/vector transformants after prolonged SM treatment for 120 min. The gradual decrease in Gcn4 abundance in *hom6Δ* cells (Fig. 4B) is consistent with the greater reduction in Gcn4 target gene transcription seen at 120 min versus 30 min of SM treatment (Fig. 1D). Moreover low-level *HIS4* mRNA was observed in the *hom6Δ HOM3^fbr^* strain even after only 30 min of SM treatment (Fig. S2).

To determine whether the reduced Gcn4 abundance on ASA accumulation reflects decreased translation of *GCN4* mRNA, we assayed a *GCN4-lacZ* fusion shown to be a faithful reporter of *GCN4* transcription and the translational efficiency of *GCN4* mRNA [33], [34]. Expression of this reporter shows the expected ∼10-fold induction in WT cells after 6 h of SM treatment, which is dampened somewhat both in the *hom6Δ* strain and in *HOM3* transformants of the *hom6Δ hom3Δ* double mutant, but not in the vector transformants of the same strain (Fig. 4C). However, the *HOM3^fbr^* and *HOM3* transformants of the double mutant exhibit indistinguishable levels of *GCN4-lacZ* expression. Similar results were obtained after only 1 h or 2 h of SM treatment (Fig. 4D), with the *hom6Δ* strain and *HOM3^fbr^* transformants of the *hom6Δ hom3Δ* double mutant both exhibiting similar reductions in *GCN4-lacZ* expression of ∼33% compared to the WT strain. As expected, a *gcn2Δ* mutant, lacking the key activator of *GCN4* mRNA translation [3], is completely defective for *GCN4-lacZ* expression (Fig. 4D). While these findings suggest a reduction in Gcn4 synthesis on ASA accumulation in cells lacking *HOM6*, the ∼33% reductions in *GCN4-lacZ* expression observed in the *hom6Δ* and *hom6Δ HOM3^fbr^* strains do not account for the 60–70% reductions in Gcn4 abundance observed after 2 h of SM treatment in the same strains. These findings suggest that Gcn4 is also degraded more rapidly than usual in response to ASA accumulation.

To provide direct evidence supporting this last conclusion, we measured the turnover of newly synthesized Gcn4 by a pulse-chase experiment. Cells were cultured with SM for 30 min and pulse-labeled with [^35^S]-methionine/cysteine for the last 15 min of the starvation period, and then chased with excess nonradioactive methionine/cysteine. Consistent with previous reports, Gcn4 is normally a highly unstable protein and decays with a half-life of ∼10–12 min in SM-treated WT cells (Fig. 4E–F) [13]. Importantly, Gcn4 decay was markedly accelerated in the *hom6Δ HOM3^fbr^* strain, with the Gcn4 half-life dropping below 5 min, thus confirming that Gcn4 is degraded more rapidly in response to ASA accumulation (Fig. 4E–F).

### Rapid degradation on ASA accumulation does not require UAS binding by Gcn4

**Figure 5:**
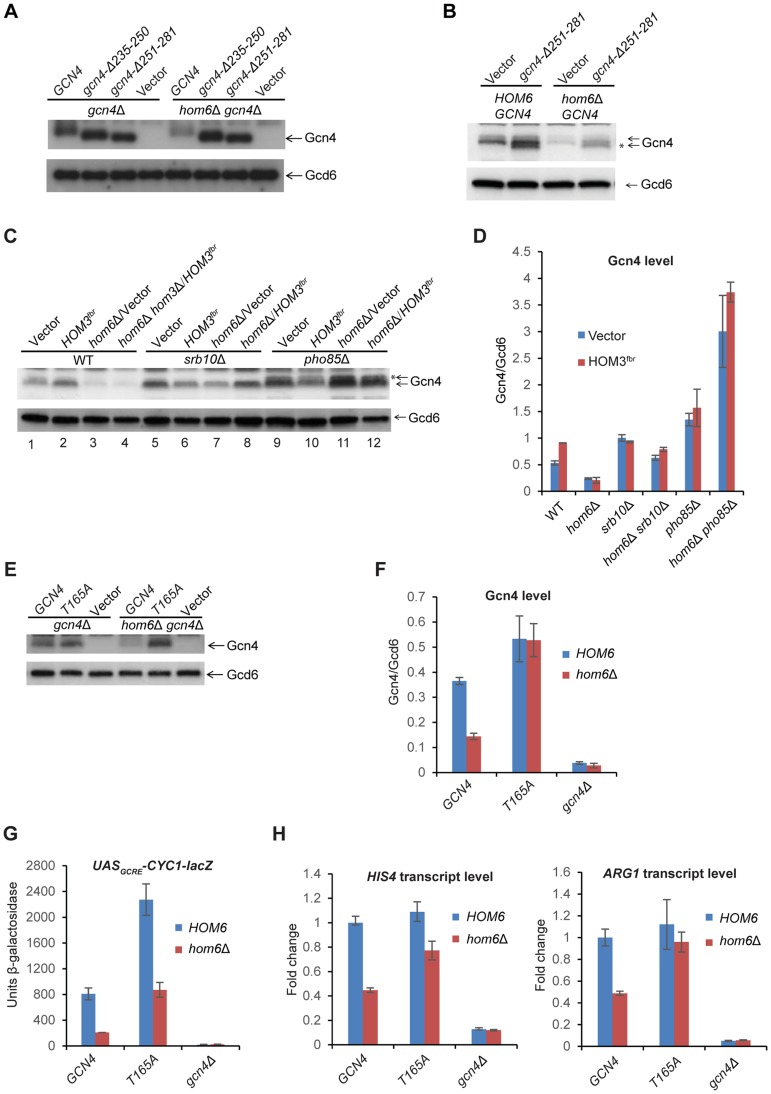
The DNA binding domain is dispensable, but CDK phosphorylation site Thr-165 is required, for depletion of Gcn4 in *hom6Δ* cells treated with SM. (A) *gcn4Δ* (F731) and *hom6Δ gcn4Δ* (YR009) strains transformed with sc plasmids with WT *GCN4* (p164), mutant alleles *gcn4-Δ235-250* (pCD114-1) or *gcn4-Δ251-281* (pCD115-1), or empty vector (YCplac33), were subjected to Western analysis as in Fig. 4A, after SM treatment for 2 h. (B) WT (BY4741) and *hom6Δ* (YR001) strains transformed with vector YCplac33 or sc mutant allele *gcn4-Δ251-281* (pCD115-1) were analyzed as in (A). * indicates mutant gcn4-*Δ*251-281 protein, displaying slightly greater electrophoretic mobility than WT Gcn4. (C) WT (BY4741), *hom6Δ* (YR001), *hom6Δ hom3Δ* (YR003), *srb10Δ* (F736), *hom6Δ srb10Δ* (YR004), *pho85Δ* (F947) and *hom6Δ pho85Δ* (YR006) strains transformed with vector (pRS313) or *HOM3^fbr^* plasmid pYPR030; were analyzed as in (A). * indicates putative phosphorylated isoforms of Gcn4. In Fig. S5, we verified that the level of Gcn4 measured by Western analysis is indistinguishable between the *hom6Δ hom3Δ HOM3^fbr^* strain examined here in lane 4 and a *hom6Δ HOM3^fbr^* strain that represents the most appropriate WT control for the mutants analyzed here in lanes 8 and 12, as would be expected from the dominance of *HOM3^fbr^*. (D) Western signals from (C) were quantified and the mean and S.E.M. Gcn4/Gcd6 ratios were calculated from 3 independent transformants. (E) *gcn4Δ* (F731) and *hom6Δ gcn4Δ* (YR009) strains transformed with sc plasmids harboring WT *GCN4* (pYPR013), *gcn4-T165A* (pYPR047), or vector (YCplac111) were analyzed as in (A). (F) Western signals from (E) were analyzed as in (D). (G) Transformants of the strains in (E) harboring pHYC2 were analyzed for *UAS_GCRE_-CYC1-lacZ* expression as in Fig. 1C. Means and S.E.Ms were calculated from three independent transformants of each strain. (H) Strains in (E) were analyzed for *HIS4* and *ARG1* mRNA levels as in Fig. 1D, after SM treatment for 2 h. Mean and S.D. values determined from two independent cultures were plotted relative to the value determined for WT cells.

As noted above, rapid degradation of Gcn4 is dependent on its phosphorylation by Pho85 and Srb10 in the nucleus, leading to its ubiquitylation and degradation by the proteasome [3]. It was also shown that the DNA-binding activity of Gcn4 is required for its sumoylation [17]. While it has been assumed that phosphorylation of Gcn4 by Srb10 likewise requires its binding to the UAS_GCRE_ [35], this has not been directly demonstrated. We hypothesized that the increased rate of Gcn4 turnover on ASA accumulation results from its increased phosphorylation by Pho85 or Srb10 and attendant degradation by the proteasome; and wished to determine whether, like sumoylation, the increased phosphorylation occurs when Gcn4 is bound to the UAS_GCRE_. To this end, we asked whether inactivating the DNA-binding ability of Gcn4 would suppress the effect of ASA accumulation on its abundance by conducting Western analysis of Gcn4 variants described previously [36] lacking the C-terminal basic region or leucine zipper, which are both required for DNA binding by Gcn4 [37]. The variant lacking the DNA binding domain, *gcn4-Δ235-250*, also lacks one of two nuclear localization sequences (NLS2) identified in Gcn4, whereas the variant lacking the leucine zipper, *gcn4-Δ251-281*, retains both NLSs, and it was shown that the leucine zipper is dispensable for nuclear localization of GFP-tagged Gcn4 [6]. We verified that both *gcn4* alleles are indistinguishable from deletion of the entire *GCN4* coding sequence in the inability to permit growth on SM-containing medium (Fig. S3). Western analysis of SM-treated cells revealed that both variants differ dramatically from WT Gcn4 and display no detectable reduction in abundance in *hom6Δ* cells treated for 2 h with SM (Fig. 5A). (Note that both truncated variants are well expressed and show the expected increased electrophoretic mobility compared to WT Gcn4 (Fig. 5A)). Furthermore, introducing *HOM3^fbr^* into *hom6Δ* cells, which severely diminishes WT Gcn4 after only 30 min of SM treatment (Fig. 4A), has no effect on abundance of the *gcn4-Δ235-250* and *gcn4-Δ251-281* mutant proteins (Fig. S4A). While these results suggested that ASA evokes accelerated degradation only when Gcn4 is capable of UAS_GCRE_-binding, it was possible that the greater stability of the mutant variants results from a failure to accumulate ASA on SM treatment owing to the absence of Gcn4-mediated derepression of threonine biosynthetic enzymes required for ASA production.

To address this last possibility, we repeated the experiment with the *gcn4-Δ251-281* strains containing or lacking *HOM6* after introducing WT *GCN4* to reinstate the GAAC. Now we observed that the abundance of the truncated *gcn4-Δ251-281* product was strongly reduced in *hom6Δ* cells, mirroring the behavior of full-length WT Gcn4 present in the same cells (Fig. 5B). The same results were observed in the corresponding strains also containing *HOM3^fbr^* (Fig. S4B). These results indicate that UAS-binding by Gcn4 is not required for its rapid degradation on ASA accumulation. Considering that Pho85 is responsible, whereas UAS-binding is dispensable, for the bulk of Gcn4 turnover under normal growth conditions [6], [12], [14], these findings are consistent with the possibility that Pho85 plays a prominent role in the accelerated degradation of Gcn4 evoked by excess ASA.

### Srb10 and Pho85 are required for the reduced abundance of Gcn4 evoked by ASA accumulation in *hom6Δ* cells

We sought next to determine the contributions of Srb10 and Pho85 to the enhanced degradation of Gcn4 in response to excess ASA. Consistent with previous findings [12], [14], deletion of either *SRB10* or *PHO85* increases the abundance of Gcn4 in otherwise WT cells treated with SM, with *pho85Δ* evoking a somewhat greater increase than *srb10Δ* (Fig. 5C, lanes 1,5,9; Fig. 5D, vector transformants). As already shown above, Gcn4 abundance is severely diminished after 120 min of SM treatment in *hom6Δ* or *hom6Δ HOM3^fbr^* cells compared to the isogenic *HOM6* cells (Fig. 5C, lanes 3–4 vs. 1–2). Importantly, eliminating *SRB10* almost completely eliminates this reduction in Gcn4 abundance in both *hom6Δ* and *hom6Δ HOM3^fbr^* cells treated with SM (Fig. 5C, lanes 7–8 vs. 3–4; & Fig. 5D). The slower migrating Gcn4 species evident in WT cells is considerably reduced in *srb10Δ* cells, suggesting that it represents a phosphorylated form of Gcn4 that depends on Srb10, which is generally consistent with previous results [14].

Deletion of *PHO85* also suppresses the reduction in Gcn4 abundance evoked by SM treatment of *hom6Δ* or *hom6Δ HOM3^fbr^* cells (Fig. 5C, cf. lanes 3–4 vs. 11–12). In fact in *pho85Δ* strains, Gcn4 abundance is higher in *hom6Δ* and *hom6Δ HOM3^fbr^* cells (where ASA accumulates) compared to the isogenic *HOM6* cells (Fig. 5C, cf. lanes 11–12 vs. 9–10; and Fig. 5D). Interestingly, deleting *PHO85* seems to increase the relative abundance of the slower migrating Gcn4 species, which presumably represent products of Srb10 phosphorylation that are not efficiently cleared in *pho85Δ* cells (Fig. 5C, cf. lanes 9–12 vs. 1–4). The results in Figs. 5C–D suggest that both Srb10 and Pho85 are required for the strong depletion of Gcn4 that occurs on ASA accumulation. The stronger effect of *pho85Δ* versus *srb10Δ* on Gcn4 abundance observed on ASA accumulation in these experiments is consistent with previous results indicating a relatively greater contribution of Pho85 to Gcn4 degradation under normal growth conditions [12], [14].

Gcn4 was found to be phosphorylated in vitro by Srb10 on multiple CDK consensus sites, including Ser17, Ser210, Thr61, Thr105 and possibly Thr165 [14], and by Pho85 both in vivo and in vitro on Thr165 [12]. Moreover, the T165A substitution alone was sufficient to confer marked stabilization of Gcn4 in vivo [12]. Importantly, we found that the Gcn4-T165A variant showed no reduction in abundance in SM-induced *hom6Δ* cells compared to *HOM6* cells (Fig. 5E–F). Moreover, replacing WT *GCN4* with the *GCN4-T165A* allele in *hom6Δ* cells restored *UAS_GCRE_-CYC1-lacZ* reporter (Fig. 5G) and *ARG1* mRNA expression (Fig. 5H) to levels essentially equivalent to those seen in *HOM6 GCN4* cells. Expression of *HIS4* mRNA also was boosted by *GCN4-T165A* in *hom6Δ* cells, although expression remained below that seen in *HOM6 GCN4* cells (Fig. 5H), suggesting either that the Gcn4-T165A variant is not functionally equivalent to WT Gcn4 or that a fraction of Gcn4-T165A rescued in *hom6Δ* cells has a lower than WT specific activity. In any event, these findings provide strong evidence that phosphorylation of T165 by Pho85 and/or Srb10 is required for the pronounced depletion of Gcn4 evoked by ASA accumulation in *hom6Δ* cells.

### Evidence that Srb10 and Pho85 eliminate functionally distinct pools of Gcn4 on ASA accumulation

Having found that removing either Srb10 or Pho85 restores high-level Gcn4 abundance during ASA accumulation in *hom6Δ* cells, we expected to find that transcriptional activation by Gcn4 would likewise be restored in both *hom6Δ srb10Δ* and *hom6Δ pho85Δ* strains, particularly since these CDKs have been implicated in reducing Gcn4 activation function via phosphorylation of Gcn4 [19]. However, we observed distinct differences in the activation function of Gcn4 in cells lacking Srb10 versus Pho85.

**Figure 6:**
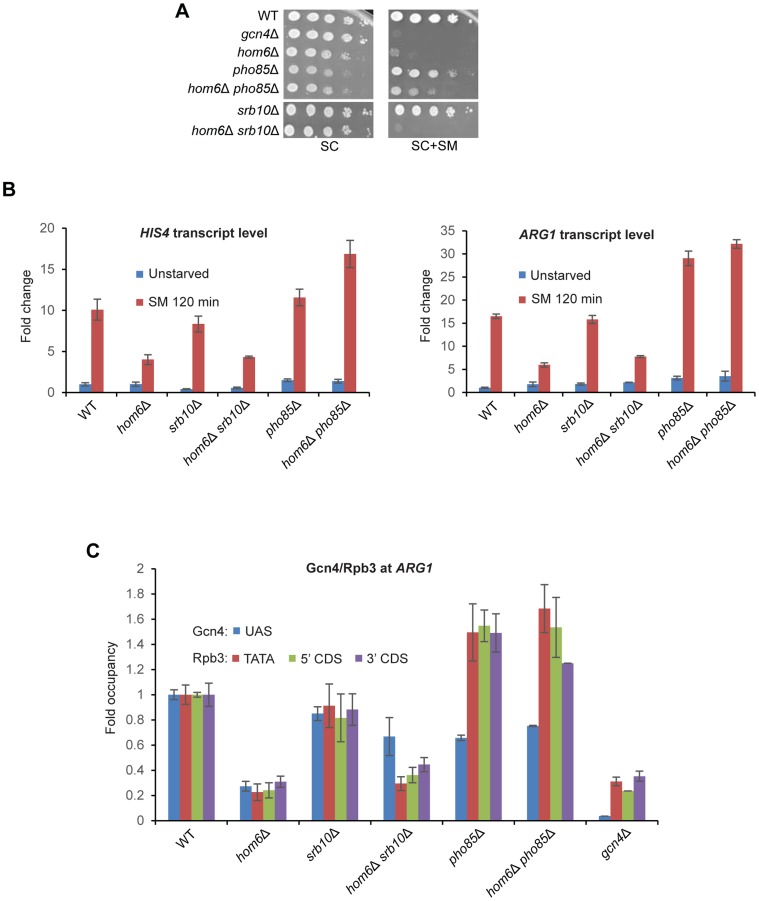
Deleting *SRB10* or *PHO85* stabilizes functionally distinct populations of Gcn4 in *hom6Δ* cells treated with SM. (**A**) WT (BY4741), *gcn4Δ* (F731), *hom6Δ* (YR001), *pho85Δ* (F947), *hom6Δ pho85Δ* (YR006), *srb10Δ* (F736) and *hom6Δ srb10Δ* (YR004) strains were analyzed as in Fig. 1B, except that the plates were incubated for 4d for SC+SM versus 2d for SC. Results for SC or SC+SM conditions derive from images of the same plate. (**B**) Yeast strains from (A) were analyzed for *HIS4* and *ARG1* mRNA levels in unstarved cells and after 120 min of SM starvation, as in Fig. 1D. Mean and S.D. values determined from two independent cultures are plotted. (**C**) Chromatin immunoprecipitation analysis of strains from (A) starved with SM for 120 min and crosslinked with formaldehyde. Sheared chromatin was immunoprecipitated with antibodies against Gcn4 or PolII subunit Rpb3, and DNA extracted from the immunoprecipitates and equivalent input samples was analyzed by quantitative PCR to determine levels of the *ARG1 UAS* for Gcn4; *ARG1* promoter (TATA) and coding sequences (5′CDS/3′CDS) for Rpb3; and non-coding sequences on chromosome V (ChrV) as a negative control. Mean and S.D. occupancies of Gcn4/Rpb3 were calculated as (target_IP_/ChrV_IP_)/(target_Input_/ChrV_Input_) from duplicate PCR amplifications of DNA samples from immunoprecipitations of chromatin prepared from two independent cultures of each strain and expressed relative to those measured for the WT strain.

First, we found that eliminating *PHO85* restores the ability of *hom6Δ* cells to grow on SM containing plates (Fig 6A). By contrast, *hom6Δ srb10Δ* cells cannot grow on SM medium, even though *HOM6 srb10Δ* cells grow at the WT rate on SM medium (Fig. 6A). These findings suggest that deletion of *SRB10* does not rescue the defective GAAC response to SM treatment in *hom6Δ* cells, whereas deletion of *PHO85* does. Consistent with the growth assays, we found that eliminating *PHO85* fully restores transcriptional activation of *HIS4* and *ARG1* in *hom6Δ* cells, conferring even higher than WT levels of both transcripts in the *hom6Δ pho85Δ* double mutants (Fig. 6B). It is noteworthy that deleting *HOM6* provokes no reduction in *HIS4* or *ARG1* mRNAs, and even seems to elevate *HIS4* mRNA, in *pho85Δ* cells (Fig. 6B). By contrast, deleting *SRB10* evokes little or no increase in *HIS4* or *ARG1* mRNA levels in SM-treated *hom6Δ* cells (Fig. 6B). The failure of *srb10Δ* to rescue activation of these genes in *hom6Δ* cells cannot be attributed simply to the loss of a coactivator function of Srb10 [10], as *srb10Δ* had little or no effect on levels of *HIS4* or *ARG1* mRNAs in otherwise WT *HOM6* cells (Fig. 6B, *srb10Δ* vs. WT), nor on the ability to grow in SM medium (Fig. 6A, *srb10Δ* vs. WT). These findings suggest that the Gcn4 molecules rescued from accelerated degradation on ASA accumulation by elimination of Srb10 are relatively nonfunctional in transcriptional activation. By contrast, the Gcn4 molecules rescued from degradation by elimination of Pho85 from *hom6Δ* cells appear to include highly functional species capable of evoking a greater than WT level of transcriptional activation.

Having found above that deleting *PHO85* restores a higher level of Gcn4 in *hom6Δ* cells than does deleting *SRB10* (Fig. 5D), it was important to determine whether the higher level of transcriptional activation seen in *hom6Δ pho85Δ* versus *hom6Δ srb10Δ* cells (Figs. 6A–B) arises simply from relatively greater UAS occupancy by Gcn4 in *hom6Δ pho85Δ* cells. To address this possibility, we conducted ChIP analysis to measure the occupancy of Gcn4 at the *ARG1* UAS and the occupancies of Rpb3 (a PolII subunit) at the promoter (TATA element) and the 5′ or 3′ ends of the CDS at *ARG1* after 2 h of SM treatment. It was shown previously that SM treatment of WT cells evokes large increases in occupancies of Gcn4 and Rpb3 at *ARG1* that are completely absent in *gcn4Δ* cells [9], [10]. Importantly, these increases in occupancy are strongly diminished in SM-treated *hom6Δ* cells (Fig. 6C), providing direct evidence that the GAAC defect in the *hom6Δ* mutant results from low-level Gcn4 occupancy of the UAS with attendant reduced recruitment of PolII to the promoter.

As expected from the ability of *srb10Δ* to restore cellular Gcn4 abundance in *hom6Δ* cells (Fig. 5C–D), Gcn4 occupancy of the *ARG1* UAS is substantially higher in *hom6Δ srb10Δ* versus *hom6Δ SRB10* cells (Fig. 6C, blue bars). However, this increase in Gcn4 occupancy is associated with much smaller increases in Rpb3 occupancies at all three locations at *ARG1* (Fig. 6C, orange, green, purple bars), consistent with the idea that the Gcn4 recovered in *hom6Δ srb10Δ* cells is relatively inactive.

Deletion of *PHO85* had strikingly different consequences on Gcn4 activity. In *HOM6* cells, the *pho85Δ* mutation evokes a reduction in UAS occupancy of Gcn4, but actually increases Rpb3 occupancies compared to the WT strain (Fig. 6C, *pho85Δ* vs. WT), which is consistent with the higher than WT levels of *ARG1* mRNA in *pho85Δ* cells shown above (Fig. 6B). This effect of *pho85Δ* was noted previously [17], and is not understood mechanistically; however, it might indicate that Pho85 clears fully functional Gcn4 molecules as a homoeostatic mechanism to prevent hyperinduction of the GAAC response, such that UAS-bound Gcn4 has a greater than WT specific activity in *pho85Δ* cells.

Despite the much higher total cellular abundance of Gcn4 observed in *hom6Δ pho85Δ* versus *hom6Δ srb10Δ* cells (Fig. 5D), Gcn4 occupancy of the *ARG1* UAS is comparable in these two strains (Fig. 6C, blue bars). In contrast, the Rpb3 occupancies at all three locations at *ARG1* are substantially higher in the *hom6Δ pho85Δ* versus *hom6Δ srb10Δ* cells (Fig. 6C, orange, green, purple bars). In fact, the Rpb3 occupancies observed in *hom6Δ pho85Δ* cells exceed those in WT cells despite a lower than WT level of Gcn4 UAS occupancy in the mutant cells (Fig. 6C). These findings suggest that the Gcn4 molecules rescued in *hom6Δ pho85Δ* cells that are capable of UAS binding have a greater than WT specific activity.

As elaborated in the Discussion, the reduced ability of UAS-bound Gcn4 to activate transcription in the *hom6Δ srb10Δ* double mutant could be explained by proposing that Gcn4 is rendered less functional in response to ASA accumulation and that Srb10 is required to clear the inactive Gcn4 molecules from the promoter by targeting them for degradation. The apparent hyperactivity of UAS-bound Gcn4 in *hom6Δ pho85Δ* cells could be explained by proposing that Pho85 targets both fully functional Gcn4 and defective species rendered incapable of UAS-binding on ASA accumulation in *hom6Δ* cells.

### Evidence that ASA accumulation increases sumoylation of Gcn4

**Figure 7:**
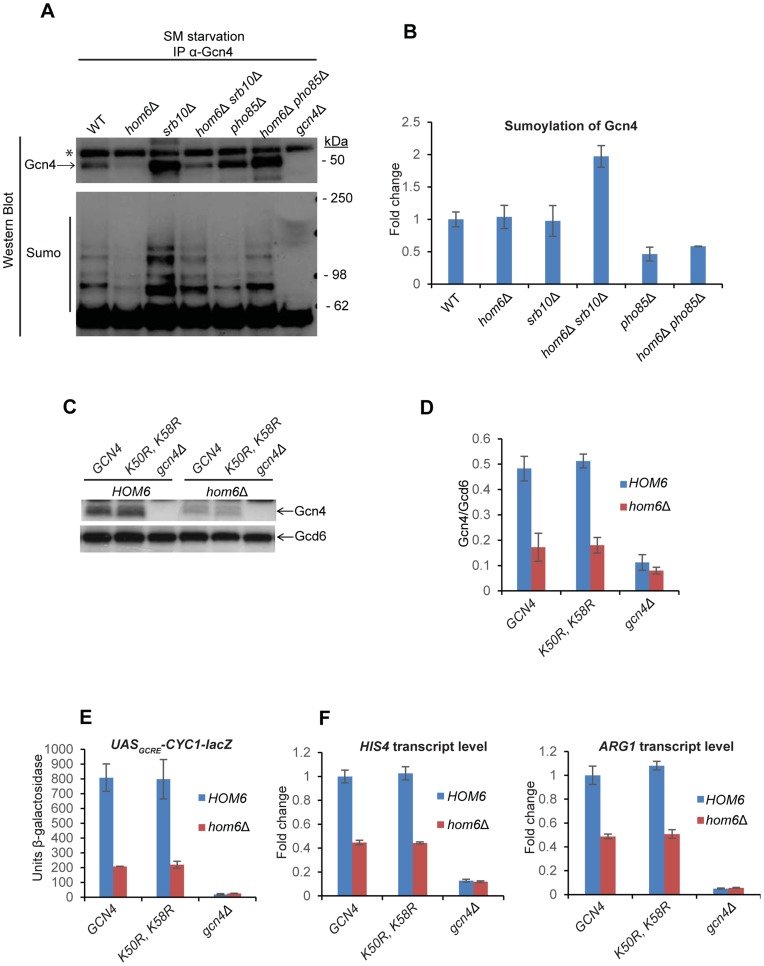
Gcn4 is hypersumoylated in *hom6Δ srb10Δ* cells treated with SM. (**A**) Yeast strains from Fig. 6A were treated with 0.5 µg/ml SM for 120 min and aliquots of WCE containing 1 mg total protein were immunoprecipitated with α-Gcn4 antibodies. Immunocomplexes were subjected to Western analysis with α-Gcn4 and α-Smt3 (sumo) antibodies. Relevant molecular weight markers (SeeBlue Pre-Stained Standard) are indicated. * indicates a nonspecific band observed even in the *gcn4Δ* strain. Gcn4 Western signals do not necessarily reflect the relative Gcn4 levels in the starting WCEs presumably owing to variability in the efficiency of immunoprecipitating Gcn4. (**B**) Western signals for sumoylated Gcn4 from the lower panel of (A) were normalized to the Gcn4 signals from the upper panel of (A) and the Mean and S.D. ratios calculated from two independent cultures were expressed relative to that measured for WT cells. (**C**) *gcn4Δ* (F731) and *hom6Δ gcn4Δ* (YR009) strains transformed with sc plasmids harboring WT *GCN4* (pYPR013), *gcn4-K50R, K58R* (pYPR038), or vector (YCplac111) were analyzed as in Fig. 5A. (**D**) Western signals from (C) were analyzed as in Fig. 5D. (**E**) Transformants of the strains in (C) harboring pHYC2 were analyzed for *UAS_GCRE_-CYC1-lacZ* expression as in Fig. 1C. Means and S.E.Ms were calculated from three independent transformants of each strain. (**F**) Strains in (C) analyzed for *HIS4* and *ARG1* mRNA levels as in Fig. 5H. Mean and S.D. values determined from two independent cultures are plotted.

It was shown recently that Gcn4 is sumoylated at target gene promoters, and Srb10 was implicated in clearing these sumoylated Gcn4 molecules [17]. We considered the possibility that sumoylation of Gcn4 bound to promoters increases on ASA accumulation and enhances the clearance of inactive Gcn4 by Srb10. If so, we would expect to find elevated sumoylation of Gcn4 in *srb10Δ hom6Δ* strains, but not in *pho85Δ hom6Δ* strains. To examine this possibility, we immunoprecipitated Gcn4 from whole cell extracts (WCEs) and probed the immune complexes with antibodies against Smt3 (yeast SUMO). After normalizing the Smt3 signal for Gcn4 abundance in the immune complexes, we observed that the Gcn4 present in *hom6Δ srb10Δ* cells after 2 h of SM treatment has an ∼2-fold higher level of sumoylation than observed in WT or *hom6Δ* cells under the same conditions, whereas sumoylation of Gcn4 is ∼2-fold lower in *hom6Δ pho85Δ* compared to WT or *hom6Δ* cells (Fig. 7A–B).

The finding that sumoylated Gcn4 is elevated specifically in the *srb10Δ hom6Δ* strain supports the idea that sumoylation of Gcn4 increases during SM-starvation of *hom6Δ* cells and that Srb10 targets the sumoylated Gcn4 for clearance from target promoters. As a result of Srb10 function, the proportion of Gcn4 that is sumoylated should not increase in response to ASA accumulation in *hom6Δ SRB10* cells, as we observed (Fig. 7B). By the same token, the fact that elimination of *PHO85* from *hom6Δ* cells does not significantly alter the sumoylation of Gcn4 following SM treatment implies that Pho85 plays little role in clearing sumoylated Gcn4 and is therefore restricted primarily to clearing unsumoylated Gcn4, which would include Gcn4 molecules not bound to the UAS_GCRE_.

It was shown that sumoylation of Gcn4 at Lys50 and Lys58 contributes to clearing Gcn4 from the UAS via Srb10 phosphorylation in the early stages of SM induction; and this process is eliminated by arginine substitutions at both Lys residues [17]. To determine whether sumoylation of Gcn4 stimulates the clearing of Gcn4 from promoters on ASA accumulation, we examined the effects of the K50R and K58R substitutions on Gcn4 abundance in SM-treated *hom6Δ* cells. We found that the Gcn4-K50R, K58R mutant displayed a reduction in abundance on SM-treatment of *hom6Δ* cells very similar to that observed for WT Gcn4 (Fig. 7C–D). The *K50R, K58R* substitution also had no effect on Gcn4 abundance in SM-treated *pho85Δ* and *pho85Δ hom6Δ* cells (Fig. S6), where turnover of Gcn4 is dependent on Srb10, thus suggesting that Srb10-dependent degradation of Gcn4 on ASA accumulation is not enhanced by sumoylation of Lys50/Lys58. We also found that, in otherwise WT cells, the Gcn4-K50R, K58R variant confers essentially WT SM-induction of the *UAS_GCRE_-CYC1-lacZ* reporter and *HIS4* and *ARG1* mRNAs, in accordance with previous findings [17]; and that the Gcn4-K50R, K58R variant resembles WT Gcn4 in being unable to sustain efficient SM-activation of *UAS_GCRE_-CYC1-lacZ, HIS4* and *ARG1* expression in *hom6Δ* cells (Fig. 7E–F). Thus, although our data suggest that sumoylation of promoter-bound Gcn4 increases on ASA accumulation, and that the sumoylated Gcn4 molecules are cleared from the promoter primarily by Srb10, as concluded previously [17], the sumoylation of Lys50/Lys58 is not critically required for the enhanced degradation of Gcn4 that occurs under conditions of ASA excess.

## Discussion

In this report we have shown that accumulation of ASA in *hom6* mutants lacking functional homoserine dehydrogenase (HSD) impairs the GAAC response to starvation for Ile/Val by accelerating degradation of the activator Gcn4. It is remarkable that ASA accumulation increases the rate of Gcn4 turnover considering that Gcn4 is already exceedingly short-lived under normal growth conditions [11], [12], [14]. The effect of ASA accumulation in reducing Gcn4 abundance and occupancy of the *ARG1* UAS was mitigated in mutants lacking either of the CDKs, Srb10 and Pho85, known to phosphorylate Gcn4 and target it for ubiquitylation and rapid degradation by the proteasome. Deletion of *SRB10* restored an essentially WT level of cellular Gcn4 in Ile/Val-starved *hom6Δ* cells, whereas deletion of *PH085* conferred an even greater than WT level of cellular Gcn4 in starved *hom6Δ* cells. Similarly, mutating a key phosphorylation site of Pho85 and possibly Srb10, Thr-165, also rescued WT Gcn4 abundance in Ile/Val-starved *hom6Δ* cells. These findings are consistent with the model that ASA accumulation evokes an increased rate of phosphorylation-dependent degradation of Gcn4 by the proteasome. While both Srb10 and Pho85 are required for the accelerated Gcn4 turnover, it appears that Pho85 plays the larger role—just as observed under normal growth conditions [12]. This last conclusion is consistent with our finding that UAS_GCRE_-binding by Gcn4 is dispensable for its rapid turnover on ASA accumulation, which is also the case under normal growth conditions [6].

Interestingly, the outcome on the GAAC response differed significantly depending on which of the two CDKs was eliminated in *hom6Δ* cells. On removal of Srb10 from *hom6Δ* cells, the recovery of UAS-bound Gcn4 was accompanied by only a small increase in transcriptional activation of *ARG1*, such that *srb10Δ hom6Δ* cells cannot grow on SM medium. By contrast, *hom6Δ* cells lacking Pho85 can grow on SM medium, and we observed an even greater than WT activation of *ARG1* transcription conferred by essentially the same level of UAS-bound Gcn4 seen in *hom6Δ srb10Δ* cells, which is actually less than the UAS occupancy of Gcn4 found in fully WT cells. It could be argued that the low-level activation of *ARG1* transcription seen in the *srb10Δ hom6Δ* double mutant reflects the requirement for Srb10 for efficient activation by Gcn4 observed previously [7], [10]. However, here we observed no effect of deleting *SRB10* on cell growth, and little or no effect on the induction of *ARG1* and *HIS4* mRNAs or PolII occupancy of *ARG1* CDS in otherwise WT SM-treated cells; and the small defects we observed seem inadequate to explain the nearly complete absence of increased *ARG1* and *HIS4* transcription and PolII occupancy at *ARG1* occurring in SM-treated *hom6Δ srb10Δ* cells. Hence, we favor the alternative explanation that the specific activity of the UAS-bound Gcn4 rescued by eliminating Srb10 in *hom6Δ* cells is lower than that rescued by eliminating Pho85 in *hom6Δ* cells. This in turn suggests that these CDKs target different populations of Gcn4.

The notion that Srb10 and Pho85 recognize different populations of Gcn4 also fits with our demonstration that Gcn4 is more highly sumoylated in Ile/Val-starved *srb10Δ hom6Δ* cells than in starved WT or *hom6Δ* cells, whereas Gcn4 is hypo-sumoylated in Ile/Val-starved *pho85Δ hom6Δ* cells. This finding is consistent with the previous conclusion that Srb10 is required to clear sumoylated Gcn4 from promoters [17]. Hence, we suggest that the putative population of defective Gcn4 molecules that are phosphorylated by Srb10 and subsequently degraded also tend to be hyper-sumoylated. However, we found that sumoylation of the known sites of this modification in Gcn4, Lys50/Lys58, was unimportant for the accelerated degradation of Gcn4 in Ile/Val-starved *hom6Δ* cells. Thus, while sumoylation appears to be a characteristic of Gcn4 molecules that are phosphorylated by Srb10 and subsequently cleared from the promoter, we have no evidence that sumoylation enhances the unusually rapid degradation of these Gcn4 molecules that occurs during ASA accumulation.

**Figure 8:**
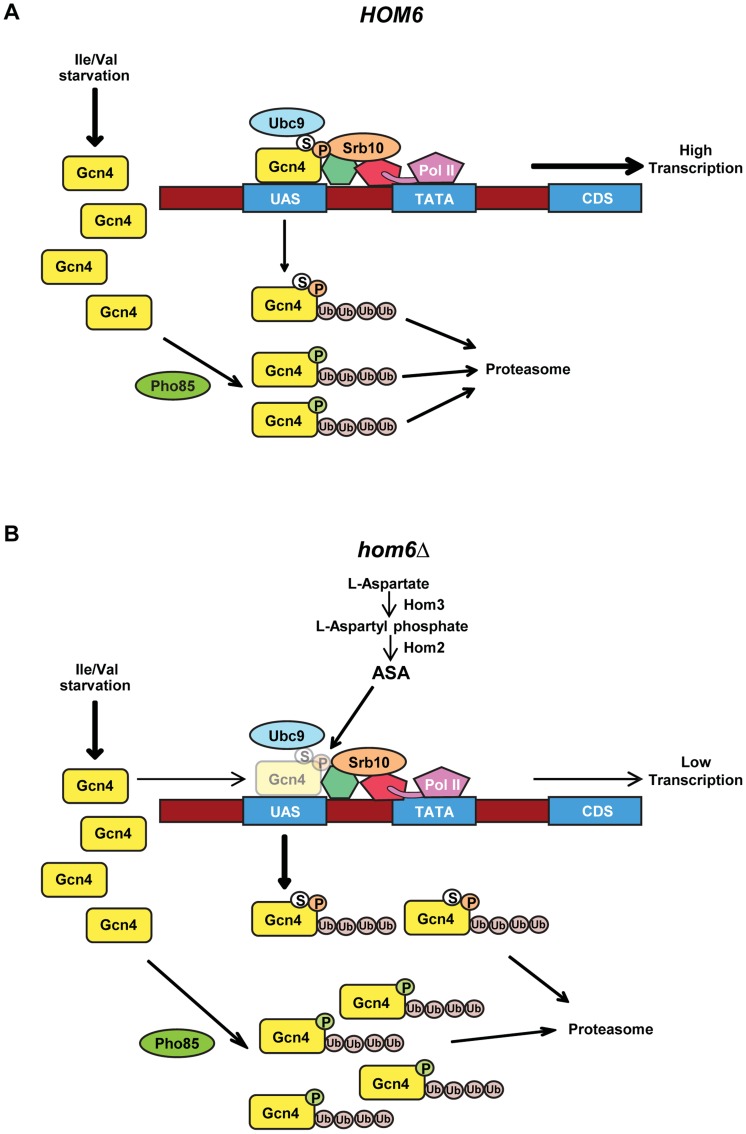
Model for the roles of Pho85 and Srb10 in accelerated turnover of Gcn4 in response to ASA accumulation in *hom6Δ* cells. (A) Starvation of WT (*HOM6*) cells for Ile/Val evokes increased synthesis of Gcn4 at the translational level and subsequent increased binding of Gcn4 to UAS elements of genes subject to GAAC. Gcn4 recruits coactivators, including Mediator (green and red shapes connecting Gcn4 to Pol II), and Pol II to the promoter (TATA) for increased transcription of target gene coding sequences (CDS). As Srb10 is associated with Mediator and recruited by UAS-bound Gcn4, we propose that Srb10 phosphorylates the bulk of UAS-bound Gcn4 molecules (orange balls labeled with “P”), stimulating their ubiquitylation and attendant degradation by the proteasome. UAS-bound Gcn4 is also sumoylated by Ubc9 (white balls labeled with “S”) and the sumoylated molecules are targeted for degradation by Srb10. Pho85 is responsible for the majority of Gcn4 turnover, and we propose it phosphorylates (green balls labeled with “P”) and triggers degradation of both active and defective non-UAS bound Gcn4 species. (B) Starvation of *hom6Δ* cells for Ile/Val also evokes increased synthesis of Gcn4 and attendant increased binding of Gcn4 to UAS elements. However, we hypothesize that ASA accumulation evokes damage or inactivating modifications of Gcn4. Damage/modification restricted to the activation domain, disengages UAS-bound Gcn4 from coactivators and increases its rate of phosphorylation by Srb10 at the promoter, with attendant increased degradation by the proteasome. (Gcn4 opacity is reduced to depict its decreased occupancy of the UAS.) Damage/modification that extends to the DNA binding or dimerization domain disengages Gcn4 from the UAS and makes it susceptible to phosphorylation by Pho85 and subsequent proteasomal degradation. Pho85 also targets functional Gcn4 molecules when they disengage from the UAS, just as in *HOM6* cells. Hence, the absence of Srb10 in *srb10Δ* cells spares from degradation defective Gcn4 molecules capable of UAS-binding and thereby reduces the specific activity of UAS-bound Gcn4. The absence of Pho85 in *pho85Δ* cells spares both fully functional Gcn4 molecules and inactive species incapable of stable UAS-binding and thereby increases the specific activity of UAS-bound Gcn4 while simultaneously decreasing the fraction of Gcn4 capable of UAS binding.

To explain in greater detail our proposal that Srb10 and Pho85 target distinct populations of Gcn4, we begin by positing that phosphorylation of the Gcn4 activation domain (AD) by Srb10 and Pho85 occurs most rapidly when the AD is not engaged with coactivators at the promoter. Hence, both functional and non-functional Gcn4 molecules not bound to the UAS would be susceptible to rapid turnover, whereas UAS-bound Gcn4 would turn over more slowly unless it harbors a damaged or modified AD that cannot engage with coactivators. Pho85 is located in the nucleus [6]; however, we have observed only low-level recruitment of Pho85 to the *ARG1* UAS_GCRE_ by ChIP analysis, at a level decidedly smaller than that seen for Srb10 (Fig. S7) or other Mediator subunits [38]. Moreover, Pho85 is responsible for the majority of Gcn4 degradation under both nonstarvation conditions and moderate-starvation conditions where the Pho85/Pcl5 complex is abundant [12], which includes our SM-induction conditions. This can explain the previous finding that Gcn4 DNA binding activity is dispensable for rapid Gcn4 turnover under such conditions [6]. Thus, we envision that Pho85 primarily targets Gcn4 molecules when they are not bound to a UAS_GCRE_. In contrast, Srb10 is recruited by Gcn4 to the *ARG1* promoter and, hence, likely plays a prominent role in the degradation of UAS-bound Gcn4 molecules that become disengaged from coactivators either stochastically or because of damage or modification of the AD (Fig. 8A). Again, this proposal is consistent with the previous finding that Srb10 is required to clear sumoylated Gcn4, as sumoylation is impaired by mutations that impair UAS-binding by Gcn4 [17].

There is evidence that Gcn4 is deactivated under normal growth conditions by Srb10 and Pho85, and that the phosphorylated, inactive protein must be degraded by the proteasome to prevent a reduction in the specific activity of UAS-bound Gcn4 [19]. We suggest that ASA accumulation in *hom6Δ* cells provokes damage or modification of Gcn4 that increases its rate of phosphorylation and subsequent turnover by the proteasome. The inability of the putative damaged or modified Gcn4 molecules to bind to the UAS or engage with coactivators could be responsible for their enhanced phosphorylation. Because eliminating the DNA binding activity of Gcn4 did not abolish its rapid turnover in *hom6Δ* cells harboring an intact GAAC, and DNA binding is not required for the Pho85-dominated turnover of Gcn4 under normal conditions [6], [12], we propose that Pho85 plays a predominant role in targeting the putative defective Gcn4 molecules generated under ASA excess, presumably when they are dissociated from the promoter; whereas Srb10 would make a lesser contribution and mediate the rapid degradation of defective, UAS-bound Gcn4 species (Fig. 8B). Accordingly, eliminating Srb10 will spare from degradation defective Gcn4 molecules that are capable of UAS binding, and because Pho85 will continue to target functional molecules when they become disengaged from the UAS, the specific activity of UAS-bound Gcn4 should decline in *srb10Δ* cells, as we observed. By contrast, eliminating Pho85 will rescue both damaged molecules incapable of UAS binding as well as functional Gcn4 molecules that are phosphorylated by Pho85 when they disengage from the UAS; and because Srb10 will continue to clear activation-defective Gcn4 species capable of UAS binding, the specific activity of UAS-bound Gcn4 should increase in *pho85Δ* cells, as we observed (Fig. 8B). Even in *HOM6 pho85Δ* cells, where no ASA accumulation occurs, the specific activity of UAS-bound Gcn4 exceeds that in WT cells, as seen both here and previously [17], and this phenomenon is also explained by our model (Fig. 8A). The proposal that Pho85 is responsible for clearing defective molecules incapable of binding to the UAS can also explain why a sizeable fraction of the Gcn4 spared from degradation in *pho85Δ* cells appears to be incapable of UAS binding, as indicated by the lower than WT UAS occupancy despite higher than WT cellular abundance of Gcn4 seen in *pho85Δ* and *pho85Δ hom6Δ* cells (Fig. 6C vs. Fig. 5C–D). As noted above, it is also possible that the lower than WT UAS-occupancy of Gcn4 in *pho85Δ* cells reflects an unknown feedback regulatory mechanism that limits Gcn4 binding to the UAS as a way to prevent hyperactivation of Gcn4 target genes beyond the elevated levels seen in *pho85Δ* cells.

An intriguing observation not anticipated by the model in Fig. 8 is that ASA accumulation provoked by SM-treatment of *hom6Δ pho85Δ* cells leads to a higher level of Gcn4 than occurs in *HOM6 pho85Δ* cells where ASA does not accumulate (Fig. 5C–D). We recently obtained evidence that most of this effect can be accounted for by an unexpected increase in *GCN4* transcription or translation, as expression of the *GCN4-lacZ* reporter was found to be ∼2-fold higher in SM-treated *hom6Δ pho85Δ* versus *HOM6 pho85Δ* cells (Fig. S8).

A final interesting question is whether attenuation of the GAAC evoked by ASA accumulation is adaptive in WT yeast in the wild. Perhaps the enzyme HSD is frequently targeted for inhibition by plants, animals, or other microorganisms as a means inhibiting yeast growth. Indeed, the threonine pathway does not exist in mammals and has been identified as a valuable target for developing new antifungal therapeutics [24]. Moreover, it was shown that a strain of *Streptomyces* produces a natural antibiotic that targets HSD [39]. Reducing threonine biosynthesis by inhibiting HSD should activate eIF2α phosphorylation by Gcn2 and thereby reduce general protein synthesis, which is an appropriate response to limitation for threonine as a means of reducing the rate of threonine consumption. However, the concurrent transcriptional induction of Gcn4 target genes, including threonine biosynthetic pathway genes, evoked by translational upregulation of *GCN4* mRNA might not be adaptive in this instance, owing to the toxic effects of ASA on cell physiology, including cytokinesis [27]. This toxicity of ASA provides a plausible rationale for the ability of this intermediate to suppress GAAC by accelerating Gcn4 turnover in the manner discovered here.

## Materials and Methods

### Media and growth conditions

Yeast strains were grown at 30°C in rich YPD medium (1% yeast extract, 2% peptone and 2% glucose) or defined synthetic complete (SC) medium (1.45g yeast nitrogen base, 5g ammonium sulfate, 2% glucose and 2g amino acid mix per liter) lacking leucine, uracil or histidine wherever appropriate for selection of plasmids; and lacking isoleucine and valine (Ile/Val) for treatment with sulfometuron (SM) at 0.5 µg/ml. Increasing threonine in SC medium from approximately 1 mM to 2.5 mM diminished the slow growth phenotype of *hom6Δ* cells, and eliminated that of *thr1Δ* and *thr4Δ* cells, in SC medium lacking Ile/Val. Therefore, overnight growth to saturation was achieved in SC medium supplemented with 2.5 mM threonine and thereafter yeast strains were cultured in SC with 1 mM threonine. Accordingly, a moderate threonine limitation was imposed in our experiments and, as threonine is a precursor in Ile/Val biosynthesis (Fig. 1A), this should intensify the limitation for Ile/Val provoked by SM treatment.

### Yeast strain construction and verification

Yeast strains used in this study are listed in Table 1. Yeast strains purchased from Research Genetics or previously reported were verified for all auxotrophic requirements indicated in the genotype; and gene deletions were confirmed by PCR amplification of predicted deletion junctions using primers described in Table S1. To generate *HOM6* deletion strains, the appropriate *hphMX4* gene deletion cassette conferring hygromycin B resistance [40] was PCR-amplified from plasmid pAG32 using primers HOM6-MX4-F and HOM6-MX4-R, thus introducing homologous flanking sequences upstream and downstream of *HOM6* coding sequences, and used to delete *HOM6* by transforming the appropriate strains to hygromycin B resistance on YPD agar plates. *HOM6* deletion was further confirmed by demonstrating acquisition of threonine auxotrophy, except when deleted in *hom2Δ* or *hom3Δ* strains F2057 and F1929, respectively; and by PCR-amplification of predicted junction fragments containing *hphMX4* and sequences upstream or downstream of *HOM6* coding sequences using primer pairs HOM6-A/HphMX-R1 and HOM6-DN-R/HphMX-F1 respectively. To generate *SRB10-myc_13_* and *PHO85-myc_13_* strains, a *myc_13_::HIS3MX6* cassette was PCR-amplified from plasmid pFA6a-13myc-HIS3MX6 using primer pairs SRB10-MYC13-F/SRB10-MYC13-R or PHO85-MYC13-F/PHO85-MYC13-R, respectively, and used to transform strains BY4741 and YR001to His^+^. Cassette insertions were confirmed by PCR analysis of genomic DNA using the appropriate primers specific for the *myc_13_::HIS3MX6* cassette and *SRB10* or *PHO85*, and by Western analysis of whole cell extracts (WCEs) using anti-Myc antibodies (Roche). To generate *GCN4* deletion strains, plasmid pHQ1240 containing a *gcn4Δ::hisG::URA3::hisG* cassette was digested with SspI and used to transform strains F947 and YR006 to Ura^+^. Deletion of *GCN4* was indicated by acquisition of SM-sensitivity and verified by PCR amplification of *gcn4Δ::hisG::URA3::hisG* from chromosomal DNA using primer pairs specific for sequences upstream and downstream of the *GCN4* CDS. The *URA3* gene was subsequently evicted by selecting for growth on medium containing 5-fluoroorotic acid.

**Table 1:** Yeast strains used in this study.

Strain name	Parent	Genotype	Source
F729/BY4741	NA^a^	*MATa his3Δ1 leu2Δ0 met15Δ0 ura3Δ0*	Research Genetics
F731	BY4741	*MATa his3Δ1 leu2Δ0 met15Δ0 ura3Δ0 gcn4Δ::kanMX4*	Research Genetics
F2057	BY4741	*MATa his3Δ1 leu2Δ0 met15Δ0 ura3Δ0 hom2Δ::kanMX4*	Research Genetics
F1929	BY4741	*MATa his3Δ1 leu2Δ0 met15Δ0 ura3Δ0 hom3Δ::kanMX4*	Research Genetics
F941	BY4741	*MATa his3Δ1 leu2Δ0 met15Δ0 ura3Δ0 hom6Δ::kanMX4*	Research Genetics
F2056	BY4741	*MATa his3Δ1 leu2Δ0 met15Δ0 ura3Δ0 thr1Δ::kanMX4*	Research Genetics
F926	BY4741	*MATa his3Δ1 leu2Δ0 met15Δ0 ura3Δ0 thr4Δ::kanMX4*	Research Genetics
F736	BY4741	*MATa his3Δ1 leu2Δ0 met15Δ0 ura3Δ0 srb10Δ::kanMX4*	Research Genetics
F947	BY4741	*MATa his3Δ1 leu2Δ0 met15Δ0 ura3Δ0 pho85Δ::kanMX4*	Research Genetics
H2931	BY4741	*MATa leu2Δ0 met15Δ0 ura3Δ0 <HIS3> gcn2Δ::hisG*	[48]
YR001	BY4741	*MATa his3Δ1 leu2Δ0 met15Δ0 ura3Δ0 hom6Δ::hphMX4*	This study
YR022	F2057	*MATa his3Δ1 leu2Δ0 met15Δ0 ura3Δ0 hom2Δ::kanMX4 hom6Δ::hphMX4*	This study
YR003	F1929	*MATa his3Δ1 leu2Δ0 met15Δ0 ura3Δ0 hom3Δ::kanMX4 hom6Δ::hphMX4*	This study
YR004	F736	*MATa his3Δ1 leu2Δ0 met15Δ0 ura3Δ0 srb10Δ::kanMX4 hom6Δ::hphMX4*	This study
YR006	F947	*MATa his3Δ1 leu2Δ0 met15Δ0 ura3Δ0 pho85Δ::kanMX4 hom6Δ::hphMX4*	This study
YR009	F731	*MATa his3Δ1 leu2Δ0 met15Δ0 ura3Δ0 gcn4Δ::kanMX4 hom6Δ::hphMX4*	This study
YR013	BY4741	*MATa his3Δ1 leu2Δ0 met15Δ0 ura3Δ0 SRB10-myc_13_::HIS3MX6*	This study
YR015	YR001	*MATa his3Δ1 leu2Δ0 met15Δ0 ura3Δ0 hom6Δ::hphMX4 SRB10-myc_13_::HIS3MX6*	This study
YR017	BY4741	*MATa his3Δ1 leu2Δ0 met15Δ0 ura3Δ0PHO85-myc_13_::HIS3MX6*	This study
YR019	YR001	*MATa his3Δ1 leu2Δ0 met15Δ0 ura3Δ0 hom6Δ::hphMX4 PHO85-myc_13_::HIS3MX6*	This study
YR048	F947	*MATa his3Δ1 leu2Δ0 met15Δ0 ura3Δ0 pho85Δ::kanMX4 gcn4Δ::hisG*	This study
YR050	YR006	*MATa his3Δ1 leu2Δ0 met15Δ0 ura3Δ0 pho85Δ::kanMX4 hom6Δ::hphMX4 gcn4Δ::hisG*	This study

a NA, not applicable.

### Plasmid constructions

All plasmids used in this study are listed in Table 2, and primers used in plasmid constructions are listed in Table S1. To construct pYPR010, *HOM6* (chrX:689,322.690,749) was PCR-amplified from chromosomal yeast DNA of strain BY4741, using primers HOM6-HindIII-F and HOM6-BamHI-R, and inserted between the *Hind*III and *Bam*HI sites in YCplac111. To construct pYPR018, pYPR020, pYPR022 and PYPR024, *HOM6* in pYPR010 was mutagenized using the QuikChange II XL Site-Directed Mutagenesis Kit (Stratagene) to produce *hom6* mutant alleles encoding the K117A, E208D, E208L and D219L substitutions, respectively, using sets of complementary primer pairs harboring the corresponding mutations (Table S1).

**Table 2:** Plasmids used in this study.

Plasmid name	Relevant description	Source
YCplac111	sc^a^ *LEU2*	[49]
pRS313	lc^b^ *HIS3*	[50]
YCplac33	sc *URA3*	[49]
p164	sc *URA3* with WT *GCN4* in YCp50	[33]
pCD114-1	sc *URA3* with *gcn4-*Δ*235-250* in YCp50	[36]
pCD115-1	sc *URA3* with *gcn4-*Δ*251-281* in YCp50	[36]
p180	sc *URA3* with *GCN4-lacZ* in YCp50	[33]
pHYC2	hc^c^ *URA3* with *UAS_GCRE_-CYC1-lacZ*	[51]
p367	lc *URA3* with *HIS4-lacZ* reporter	[52]
pAG32	*hphMX4* cassette	[40]
pFA6a-13myc-HIS3MX6	Myc_13_ tagging insertion cassette *myc_13_::HIS3MX6*	[53]
pHQ1240	*gcn4Δ::hisG::URA3::hisG* cassette	[54]
pYPR010	sc *LEU2* with WT *HOM6* inserted at *Hind*III and *Bam*HI sites of YCplac111	This study
pYPR018	sc *LEU2* with *hom6-K117A*, derived from pYPR010	This study
pYPR020	sc *LEU2* with *hom6-E208D*, derived from pYPR010	This study
pYPR022	sc *LEU2* with *hom6-E208L*, derived from pYPR010	This study
pYPR024	sc *LEU2* with *hom6-D219L*, derived from pYPR010	This study
pYPR028	lc *HIS3* with WT *HOM3* inserted at *Spe*I and *Eco*RI sites of pRS313	This study
pYPR030	lc *HIS3* with *HOM3-E282D (HOM3^fbr^*) inserted at *Spe*I and *Eco*RI sites of pRS313	This study
pYPR013	sc *LEU2* with WT *GCN4* inserted at *Sma*I and *Spe*I sites of YCplac111	This study
pYPR038	sc *LEU2* with *gcn4-K50R, K58R*, inserted at *Sph*I and *Spe*I sites of YCplac111	This study
pYPR047	sc *LEU2* with *gcn4-T165A*, inserted at *Sph*I and *Spe*I sites of YCplac111	This study

a sc, single copy.

b lc, low copy.

c hc, high copy.

To construct pYPR028, *HOM3* (chrV:256,132.258,737) was PCR-amplified from chromosomal yeast DNA of BY4741 using primers HOM3-F1 and HOM3-R1 and inserted between the *Spe*I and *Eco*RI sites in pRS313. pYPR030, containing *HOM3-E282D (HOM3^fbr^*), was generated by fusion-PCR using HOM3-F1 and HOM3-R1 as outside primers and complementary primers HOM3-E282D-F and HOM3-E282D-R encoding the appropriate mutation, and inserted between the *Spe*I and *Eco*RI sites of pRS313. To construct pYPR013, the *Apa*I-*SpeI* fragment containing *GCN4* was isolated from plasmid p164, polishing the *Apa*I end using Klenow polymerase exonuclease activity, and inserted between the *Sma*I and *Spe*I sites of YCplac111. pYPR038 and pYPR047 were constructed by fusion-PCR using Gcn4c-SphI-F and GCN4c-SpeI-R as outside primers in combination with primers GCN4-K50,58R-F and GCN4-K50,58R-R or primers GCN4-T165A-F and GCN4-T165A-R, respectively, and pYPR013 as PCR template. The PCR products were inserted between the *Sph*I and *Spe*I sites in YCplac111.

### Assaying *lacZ* reporters

Yeast strains transformed with plasmids pHYC2 (*UAS_GCRE_-CYC1-lacZ*), p367 (*HIS4-lacZ*), or p180 (*GCN4-lacZ*) were grown to saturation and diluted in two identical cultures in SC-Ura/Ile/Val at A_600_ = 0.5, and after 2.5 h of growth, 0.5 µg/ml SM was added to one set of cultures. Cells were harvested from untreated (unstarved) cultures after a total 6 h of growth and SM-treated cultures grown for 6 h in the presence of SM [7]. Whole cell extracts (WCEs) were prepared and assayed for β-galactosidase activity as previously described [41]. Mean specific activities were calculated from results obtained from three independent transformants.

### Quantification of mRNA abundance by real-time qRT-PCR

Yeast strains were cultured to an A_600_ of 0.4–0.6 in SC-Ile/Val, achieving at least two cell doublings, and treated with 0.5 µg/ml SM for the indicated times or left untreated. Total RNA was isolated by hot phenol extraction as previously described [42]. RNA concentration was quantified by Nanodrop spectroscopy and analyzed for integrity by agarose gel electrophoresis and ethidium bromide staining. An aliquot of 1 µg total RNA was used for cDNA synthesis using SuperScript III First-strand Synthesis Supermix for qRT-PCR (Invitrogen) and the resulting cDNA was diluted 10-fold. qRT-PCR was performed using Brilliant III Ultra-Fast qPCR Master Mix (Agilient Technologies) using the diluted cDNA in multiplex PCR and the appropriate TaqMan probes (Table S1) to quantify *ACT1* (labelled with FAM), *ARG1*, or *HIS4* (both labelled with HEX). qRT-PCR reactions were performed in triplicate using cDNA synthesized from RNA extracted from at least two independent cultures. *ARG1* or *HIS4* cDNA abundance was normalized to that of *ACT1* by calculating 2^(−ΔC^_t_^)^, where ΔC_t_ is (C_t (Target)_- C_t (*ACT1*)_). Fold changes in mRNA abundance were normalized to those measured in uninduced WT cells, or as indicated, and plotted.

### Chromatin immunoprecipitations (ChIP)

ChIP assays were conducted essentially as described previously [7], [43]. Yeast strains were cultured in 100 ml SC-Ile/Val as described above for RNA isolation, treated with 0.5 µg/ml SM for 2 h or as indicated, cross-linked for 15 min with 10 ml formaldehyde solution (50 mM HEPES KOH, pH 7.5, 1 mM EDTA, 100 mM NaCl and 11% formaldehyde) and quenched with 15 ml of 2.5 M glycine. WCEs were prepared by glass beads lysis in 400 µl FA lysis buffer (50 mM HEPES KOH, pH 7.5, 1 mM EDTA, 150 mM NaCl, 1% TritonX-100 and 0.1% Na-deoxycholate) with protease inhibitors for 45 min at 4°C and the supernatant collected after removing the beads was pooled with 600 µl FA lysis buffer used for washing the beads. The resulting lysate was sonicated to yield DNA fragments of 300–500 bp and cleared by centrifugation. 50 µl aliquots of lysates were immunoprecipitated for 2 h at 4°C with α-Gcn4, (Rabbit) [13] or α-Rpb3 antibodies (Mouse, Neoclone) coupled with α-rabbit IgG or α-mouse IgG conjugated magnetic beads (Dynabeads, Invitrogen), respectively, or with α-c-Myc (Rabbit, Roche) coupled with α-rabbit IgG conjugated magnetic beads. Recovered immune complexes were washed and eluted as described [43]. For matched input and IP samples, the crosslinks were reversed by incubation at 65°C overnight, treated with proteinase K, extracted twice with phenol:chloroform:isoamyl alcohol (25∶24∶1) and once with chloroform:isoamyl alcohol (24∶1), and ethanol precipitated, resuspending the resulting pellets in 30–40 µl TE containing RNAase as described earlier [43]. Quantitative PCRs were performed in the presence of [^33^P]-dATP with undiluted IP DNA and 500-fold diluted input DNA and further analyzed as previously described [7], [43]. The primers employed for ChIP analysis are listed in Table S1.

### Western blot analysis

WCEs were prepared in denaturing conditions with trichloroacetic acid, as described previously [44] and analyzed by immunoblotting with α-Gcd6 [45] and affinity purified α-Gcn4 antibodies [13]. Western signals were quantified by ImageJ software.

### Pulse-chase analysis of Gcn4 degradation

The analysis was performed essentially as previously described [13], [46]. Yeast cells collected from a 10 ml culture at A_600_ = 0.4–0.6 were washed with SC-Met/Ile/Val, inoculated into 0.5 ml SC-Met/Ile/Val containing 1 µg/ml SM and incubated for 15 min in a shaking water bath at 30°C; after which 1.0 mCi [^35^S]methionine/cysteine labelling mix was added and incubation continued for an additional 15 min. Cells were collected, transferred to 5 ml of pre-warmed SC-Ile/Val containing 10 mM methionine and 10 mM cysteine, and an 1 ml aliquots were removed immediately or after appropriate times of chase. Aliquots were denatured with 170 µl of 1.85 M NaOH, 7.4% 2-marcaptoethanol and precipitated with 70 µl 100% TCA on ice, washed with chilled acetone and dried under vacuum in a SpeedVac. The dried pellets were resuspended in 120 µl of 2.5% SDS, 5 mM EDTA, 1 mM PMSF by vortexing, boiled for 1 min, and cleared by centrifugation. Incorporation of label was measured by scintillation counting [13] and aliquots of extract containing equal amounts of radioactivity (5.7×10^5^ cpm) were combined with 1 ml of immunoprecipitation (IP) buffer (50 mM Na-HEPES [pH 7.5], 150 mM NaCl, 5 mM EDTA, 1% Triton X-100, 1 mM PMSF) containing 1 mg/ml BSA and 1 µl affinity-purified α-Gcn4 antibodies and mixed by rotating at 4°C for 2 h. Twenty µl of a 50% slurry of protein A-agarose beads pretreated with IP buffer containing BSA (1 mg/ml) was added, and mixing continued for 2 h. The beads were washed thrice with 500 µl cold IP buffer containing 0.1% SDS, resuspended in loading buffer, boiled, and resolved by SDS-PAGE using 4 to 20% gels. The gel was dried and subjected to autoradiography, and the [^35^S]-labeled Gcn4 was quantified by phosphorimaging analysis.

### Analysis of Gcn4 sumoylation

A modification of a previously described protocol was employed [47], as follows. Yeast strains were cultured and treated with SM as described above for ChIP analysis. 40–60 A_600_ units of cells were lysed at 4°C with glass beads by 10 cycles of vortexing, 30s-on and 30s-off, in 500 µL of chilled lysis buffer (50 mM Tris-HCl [pH 8.0], 5 mM EDTA, 150 mM NaCl, 0.2% Triton ×100 and 1 mM PMSF) containing 10 mM sodium ethyl maleimide (NEM) and protease inhibitors. The resulting lysate was cleared by centrifugation at 13,000 rpm for 30 min at 4°C and soluble protein concentration was determined by the Bio-Rad protein assay. For each sample, a 40 µl suspension of magnetic beads conjugated with α-Rabbit IgG (Dynabeads, Invitrogen) was washed twice with lysis buffer containing 5 mg/mL BSA and rotated with 1 µl affinity purified α-Gcn4 antibody [13] in 200 µl lysis buffer/BSA for 3 h at 4°C. The magnetic beads coupled with α-Gcn4 antibody were washed twice with lysis buffer/BSA to remove unbound antibody and resuspended in 200 µl lysis buffer/BSA. Aliquots containing 1 mg of protein were added to the magnetic beads suspension, adjusting the final volume to 500 µl with lysis buffer/BSA, and further rotated for 2 h at 4°C. IP samples were washed thrice with lysis buffer containing 0.1% SDS and resuspended in 30 µl 1× Novex tris-glycine SDS sample buffer (Invitrogen) and boiled for 3 min. Aliquots of 5 µl and 25 µl were subjected to Western analysis with α-Gcn4 antibodies [13] and α-SUMO (α-Smt3) polyclonal antibodies [47].

## Supporting Information

Figure S1 Expression of *HOM3^fbr^* in *hom6Δ* cells confers slow growth. Yeast strains described in Fig. 3D were analyzed for growth in spotting assays as in Fig 1B except for the use of SC-His medium containing 2.5 mM threonine.
(PDF)

Figure S2 Accelerated reduction in *HIS4* mRNA abundance evoked by SM treatment of *hom6Δ HOM3^fbr^* versus *hom6Δ* cells. WT (BY4741) and *hom6Δ* (YR001) strains transformed with vector (pRS313) and *hom6Δ hom3Δ* strain YR003 transformed with lc *HOM3^fbr^* plasmid pYPR030 (indicated as *hom6Δ HOM3^fbr^*) were cultured in SC-His/Ile/Val for at least two doublings to A_600_ = 0.4–0.6 and subjected to SM treatment (0.5 µg/ml) for the indicated times and analyzed for *HIS4* mRNA levels as in Fig 1D.
(PDF)

Figure S3 Truncation of the Gcn4 DNA binding domain eliminates complementation of the SM-sensitivity of *gcn4Δ* cells. A *gcn4Δ* strain (F731) transformed with sc plasmids with WT *GCN4* (p164), mutant alleles *gcn4-Δ235-250* (pCD114-1) or *gcn4-Δ251-281* (pCD115-1), or empty vector (YCplac33), were analyzed as in Fig 1B.
(PDF)

Figure S4 The DNA binding domain of Gcn4 is dispensable for its rapid depletion on ASA accumulation in *HOM3^fbr^ hom6Δ* cells. Transformants of strains described in Fig. 5A (**A**) and Fig. 5B (**B**) harboring *HOM3^fbr^* plasmid pYPR030 were analyzed as in Fig. 4A after SM treatment for 30 min. * indicates the gcn4-*Δ*251-281 variant.
(PDF)

Figure S5 Expression of *HOM3^fbr^* confers nearly identical reductions in Gcn4 abundance on SM treatment of *hom6Δ* versus *hom6Δ hom3Δ* cells. *hom6Δ* (YR001) and *hom6Δ hom3Δ* (YR003) strains transformed with vector (pRS313) or *HOM3^fbr^* plasmid pYPR030 were analyzed as in Fig. 5A.
(PDF)

Figure S6 Arginine substitutions of sumoylated Gcn4 residues Lys-50 and Lys-58 does not affect Gcn4 abundance in SM-treated *hom6*Δ cells lacking Pho85. *pho85Δ gcn4Δ* (YR048) and *hom6Δ pho85Δ gcn4Δ* (YR050) strains transformed with sc plasmids harboring WT *GCN4* (pYPR013) or *gcn4-K50R, K58R* (pYPR038) were analyzed as in Fig. 5A and Western signals were quantified and plotted as in Fig. 5D.
(PDF)

Figure S7 Greater recruitment of Srb10 versus Pho85 by Gcn4 to the *ARG1* UAS. *SRB10-myc_13_* (YR013) and *hom6Δ SRB10-myc_13_* (YR015) strains (**A**) and *PHO85-myc_13_* (YR017) and *hom6Δ PHO85-myc_13_* (YR019) strains (**B**) were tested for recruitment of Myc_13_-tagged Srb10 or Myc_13_-tagged Pho85 to the *ARG1 UAS* by chromatin immunoprecipitation, conducted as in Fig. 6C except using c-Myc antibodies, in cultures left untreated or treated with SM for 30 or 120 min.
(PDF)

Figure S8 *GCN4-lacZ* expression is induced in SM treated *hom6Δ pho85Δ* cells. WT (BY4741), *pho85Δ* (F947) and *hom6Δ pho85Δ* (YR006) strains transformed with vector (pRS313) or *HOM3^fbr^* plasmid pYPR030 and expression of the *GCN4-lacZ* reporter on p180 was measured as in Fig. 1C after SM treatment for 2 h. Means and S.E.Ms were calculated from three independent transformants of each strain.
(PDF)

Table S1 Primers used in this study.
(DOCX)
