# Supplementary figures and images for: Accumulation of a Threonine Biosynthetic Intermediate Attenuates General Amino Acid Control by Accelerating Degradation of Gcn4 via Pho85 and Cdk8
Source: PLoS Genet. 2014 Jul 31;10(7):e1004534. doi: 10.1371/journal.pgen.1004534 (PMC4117449; doi:10.1371/journal.pgen.1004534)

Figure S1

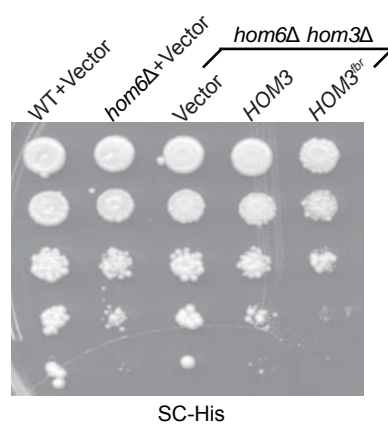

Supplement: Figure S1 — Expression of HOM3fbr in hom6Δ cells confers slow growth. Yeast strains described in Fig. 3D were analyzed for growth in spotting assays as in Fig 1B except for the use of SC-His medium containing 2.5 mM threonine. (PDF) [file pgen.1004534.s001.pdf]

Figure S2

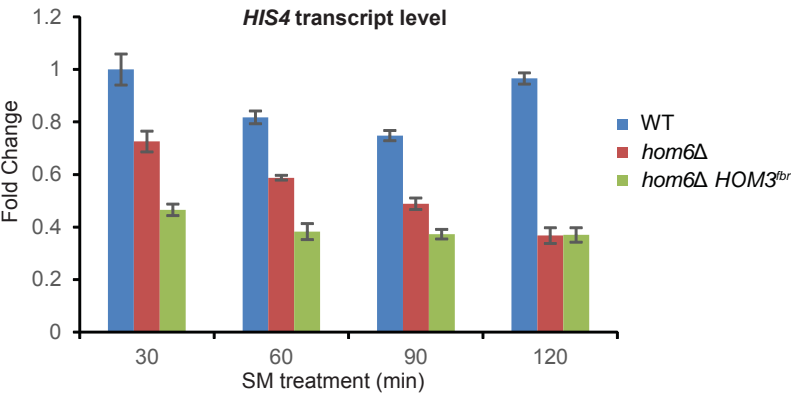

Supplement: Figure S2 — Accelerated reduction in HIS4 mRNA abundance evoked by SM treatment of hom6Δ HOM3fbr versus hom6Δ cells. WT (BY4741) and hom6Δ (YR001) strains transformed with vector (pRS313) and hom6Δ hom3Δ strain YR003 transformed with lc HOM3fbr plasmid pYPR030 (indicated as hom6Δ HOM3fbr) were cultured in SC-His/Ile/Val for at least two doublings to A600 = 0.4–0.6 and subjected to SM treatment (0.5 µg/ml) for the indicated times and analyzed for HIS4 mRNA levels as in Fig 1D. (PDF) [file pgen.1004534.s002.pdf]

Figure S3

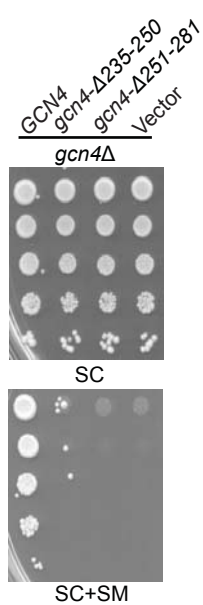

Supplement: Figure S3 — Truncation of the Gcn4 DNA binding domain eliminates complementation of the SM-sensitivity of gcn4Δ cells. A gcn4Δ strain (F731) transformed with sc plasmids with WT GCN4 (p164), mutant alleles gcn4-Δ235-250 (pCD114-1) or gcn4-Δ251-281 (pCD115-1), or empty vector (YCplac33), were analyzed as in Fig 1B. (PDF) [file pgen.1004534.s003.pdf]

Figure S4

**A**

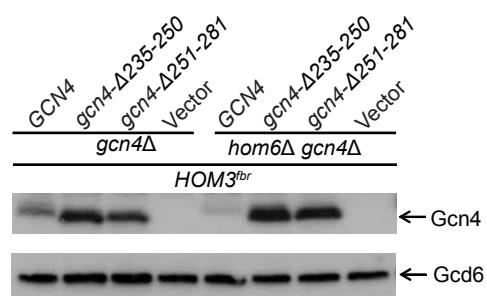

**B**

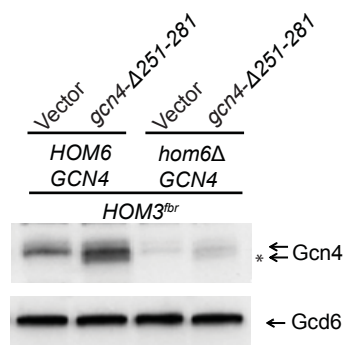

Supplement: Figure S4 — The DNA binding domain of Gcn4 is dispensable for its rapid depletion on ASA accumulation in HOM3fbr hom6Δ cells. Transformants of strains described in Fig. 5A (A) and Fig. 5B (B) harboring HOM3fbr plasmid pYPR030 were analyzed as in Fig. 4A after SM treatment for 30 min. * indicates the gcn4-Δ251-281 variant. (PDF) [file pgen.1004534.s004.pdf]

Figure S5

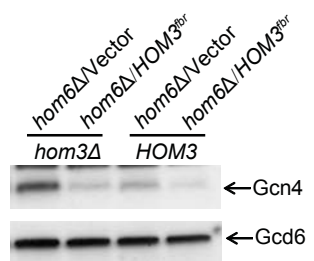

Supplement: Figure S5 — Expression of HOM3fbr confers nearly identical reductions in Gcn4 abundance on SM treatment of hom6Δ versus hom6Δ hom3Δ cells. hom6Δ (YR001) and hom6Δ hom3Δ (YR003) strains transformed with vector (pRS313) or HOM3fbr plasmid pYPR030 were analyzed as in Fig. 5A. (PDF) [file pgen.1004534.s005.pdf]

Figure S6

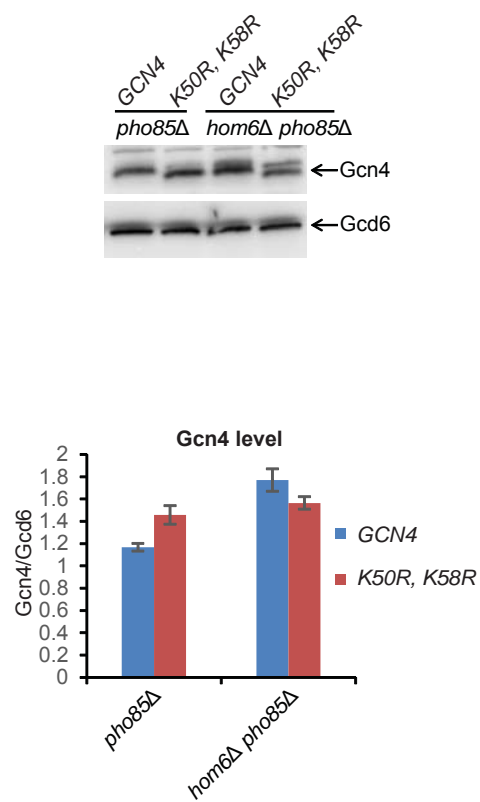

Supplement: Figure S6 — Arginine substitutions of sumoylated Gcn4 residues Lys-50 and Lys-58 does not affect Gcn4 abundance in SM-treated hom6Δ cells lacking Pho85. pho85Δ gcn4Δ (YR048) and hom6Δ pho85Δ gcn4Δ (YR050) strains transformed with sc plasmids harboring WT GCN4 (pYPR013) or gcn4-K50R, K58R (pYPR038) were analyzed as in Fig. 5A and Western signals were quantified and plotted as in Fig. 5D. (PDF) [file pgen.1004534.s006.pdf]

Figure S7

**A**

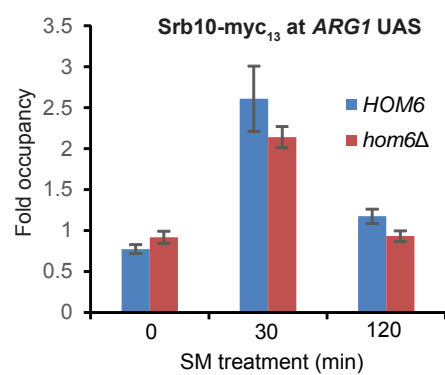

**B**

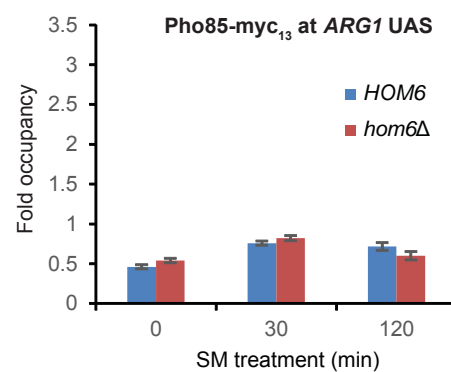

Supplement: Figure S7 — Greater recruitment of Srb10 versus Pho85 by Gcn4 to the ARG1 UAS. SRB10-myc13 (YR013) and hom6Δ SRB10-myc13 (YR015) strains (A) and PHO85-myc13 (YR017) and hom6Δ PHO85-myc13 (YR019) strains (B) were tested for recruitment of Myc13-tagged Srb10 or Myc13-tagged Pho85 to the ARG1 UAS by chromatin immunoprecipitation, conducted as in Fig. 6C except using c-Myc antibodies, in cultures left untreated or treated with SM for 30 or 120 min. (PDF) [file pgen.1004534.s007.pdf]

Figure S8

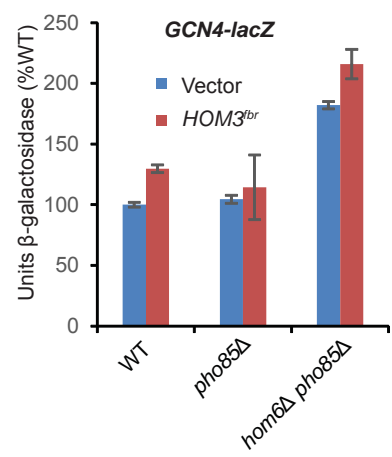

Supplement: Figure S8 — GCN4-lacZ expression is induced in SM treated hom6Δ pho85Δ cells. WT (BY4741), pho85Δ (F947) and hom6Δ pho85Δ (YR006) strains transformed with vector (pRS313) or HOM3fbr plasmid pYPR030 and expression of the GCN4-lacZ reporter on p180 was measured as in Fig. 1C after SM treatment for 2 h. Means and S.E.Ms were calculated from three independent transformants of each strain. (PDF) [file pgen.1004534.s008.pdf]
