# Supplementary material for: Accumulation of a Threonine Biosynthetic Intermediate Attenuates General Amino Acid Control by Accelerating Degradation of Gcn4 via Pho85 and Cdk8
Source: PLoS Genet. 2014 Jul 31;10(7):e1004534. doi: 10.1371/journal.pgen.1004534 (PMC4117449; doi:10.1371/journal.pgen.1004534)
Supplement: Table S1 — Primers used in this study. (DOCX) [file pgen.1004534.s009.docx]

**TABLE S1. Primers used in this study**

| Primer Name | Sequence (5'-3') | Description | Source |
| --- | --- | --- | --- |
| Primers for strain verification and construction | | | |
| KanB | CTGCAGCGAGGAGCCGTAAT | Antisense strand in *P_TEF_* sequence of *kanMX4* and *hphMX4* cassettes | [[1](#_ENREF_1)] |
| KanC | TGATTTTGATGACGAGCGTAAT | Sense strand in CDS^a^ of *KanMX4* cassette | [[1](#_ENREF_1)] |
| KanMX-R1 | GTCGCACCTGATTGCCCG | Antisense strand in CDS of *KanMX4* cassette | This study |
| HphMX-R1 | CGAGATTCTTCGCCCTCC | Antisense strand in CDS of *hphMX4* cassette | This study |
| HphMX-F1 | CGTACACAAATCGCCCGC | Sense strand in CDS of *hphMX4* cassette | This study |
| HOM2-A | AGAATTCTGACATCTGAACGTCTTC | Sense strand in sequence upstream of *HOM2* CDS | [[1](#_ENREF_1)] |
| HOM3-A | AACAGTGCTCTACTCATTGAAGGAT | Sense strand in sequence upstream of *HOM3* CDS | [[1](#_ENREF_1)] |
| HOM6-A | ACTTCTAAGCCAAGTGCAATTAAGA | Sense strand in sequence upstream of *HOM6* CDS | [[1](#_ENREF_1)] |
| HOM6-DN-R | GAATTGAGGGAAAATGGCTGCC | Antisense strand in sequence downstream of *HOM6* CDS | This study |
| SRB10-A | CAGAGACGGATTATTGTTTTCAGTT | Sense strand in sequence upstream of *SRB10* CDS | [[1](#_ENREF_1)] |
| SRB10-D | TATGTTTTAAAGCCCCATTAGTTGA | Antisense strand in sequence downstream of *SRB10* CDS | [[1](#_ENREF_1)] |
| GCN4-A | ATGGCTTGCTAAACCGATTATATTT | Sense strand in sequence upstream of *GCN4* CDS | [[1](#_ENREF_1)] |
| GCN4-DN-R | CTATTCCTAACCAGTAAATACC | Antisense strand in sequence downstream of *GCN4* CDS | This study |
| GCN2-A | TAGGAAGCAGTTGAGTAGCTGATTT | Sense strand in sequence upstream of *GCN2* CDS | [[1](#_ENREF_1)] |
| PHO85-UP-F | GCTACTTCTATCTATCCTAG | Sense strand in sequence upstream of *PHO85* CDS | This study |
| PHO85-DN-R | CTTGGGAAGCATTCACCGATC | Antisense strand in sequence downstream of *PHO85* CDS | This study |
| MYC13-HISMX-R | GGCGCGAATTCACTAGTGATTG | Antisense strand in *myc_13_::HIS3MX6* | This study |
| SRB10-CDS-F | CAAGCATGCTTTAAGCTTAC | Sense strand in *SRB10* CDS | This study |
| PHO85-CDS-F | AGACTTTATGACGTTATTC | Sense strand in *PHO85* CDS | This study |
| HOM6-MX4-F | GACTGTTTTAAGAACGTAGATAGTATCATCAATCGAATAATAAAAAAAAAATGGGATCCCCGGGTTAATTAAG | Sense strand for deleting *HOM6* using *hphMX4* cassette | This study |
| HOM6-MX4-R | ATCTATTTATATATAAATATACCTATGTTTTTATATGTCTGTTTACTGATTCATCGATGAATTCGAGCTC | Antisense strand for deleting *HOM6* using *hphMX4* cassette | This study |
| SRB10-MYC13-F | AGCAGCAGCCGCTGCTGCGGTGTCAGGAAACAATGCATCAGATGAGCCATCTCGAAAGAAAAACAGAAGACGGATCCCCGGGTTAATTAA | Sense strand for inserting *myc_13_::HIS3MX6* in 3’ end of *SRB10*  CDS | This study |
| SRB10-MYC13-R | AAAGTTTAATGAATATAATAGTGACAGTGCTGTGGAATGAAAAATTCCAAATATATATAAAAATAGAAGCGAATTCGAGCTCGTTTAAAC | Antisense strand for inserting *myc_13_::HIS3MX6* in 3’ end of *SRB10*  CDS | This study |
| PHO85-MYC13-F | GATATGAGGCTGAGCGCCAAGCAGGCTCTGCATCACCCTTGGTTTGCAGAGTACTACCACCACGCTTCACGGATCCCCGGGTTAATTAA | Sense strand for inserting *myc_13_::HIS3MX6* in 3’ end of *PHO85*  CDS | This study |
| PHO85-MYC13-R | GACTAAAGTAATAGTAGTGTATTATAGATGATTATTATCATTATATATACATGGCTACGGTTTTTCGCTGACGGGCTGCGGAATTCGAGCTCGTTTAAAC | Antisense strand for inserting *myc_13_::HIS3MX6* in 3’ end of *PHO85*  CDS | This study |
| Primers for cloning and site-directed mutagenesis | | | |
| HOM6-BamHI-R | CTGAGTGGATCCGTAGCCCCATGACATGGATGAG | Antisense strand in sequence downstream of *HOM6* CDS with *Bam*HI site. | This study |
| HOM6-HindIII-F | CTGAGTAAGCTTGTTTTTCAATAACGCACATGG | Sense strand in sequence upstream of *HOM6* with *Hind*III site | This study |
| HOM6-F2 | GCCATGAAGTCTACCATTAC | Sense strand in *HOM6* CDS | This study |
| HOM6-F3 | TCAAATTCTCTGATGTTGTC | Sense strand in *HOM6* CDS | This study |
| HOM6-K117A-F | TATTTCCATTGCTACTCCAAACGCGAAGGCCTTTTCCTCTGATTTG | Sense strand for SDM^b^ to produce *HOM6-K117A* | This study |
| HOM6-K117A-R | CAAATCAGAGGAAAAGGCCTTCGCGTTTGGAGTAGCAATGGAAATA | Antisense strand for SDM to produce *HOM6-K117A* | This study |
| HOM6-E208D-F | TCAAAGTTGCTAAAAAATTGGGTTATACTGATCCAGATCCAAGAGATG | Sense strand for SDM to produce *HOM6-E208D* | This study |
| HOM6-E208D-R | CATCTCTTGGATCTGGATCAGTATAACCCAATTTTTTAGCAACTTTGA | Antisense strand for SDM to produce *HOM6-E208D* | This study |
| HOM6-E208L-F | GTTGTCAAAGTTGCTAAAAAATTGGGTTATACTCTACCAGATCCAAGAGATG | Sense strand for SDM to produce *HOM6-E208L* | This study |
| HOM6-E208L-R | CATCTCTTGGATCTGGTAGAGTATAACCCAATTTTTTAGCAACTTTGACAAC | Antisense strand for SDM to produce *HOM6-E208L* | This study |
| HOM6-E219L-F | CAAGAGATGATTTGAATGGGTTGCTTGTTGCTAGAAAGGTTACCATTG | Sense strand for SDM to produce *HOM6-D219L* | This study |
| HOM6-E219L-R | CAATGGTAACCTTTCTAGCAACAAGCAACCCATTCAAATCATCTCTTG | Antisense strand for SDM to produce *HOM6-D219L* | This study |
| HOM3-F1 | TAGAGCTCACTAGTGCTGATCGATTCAATCGGC | Sense strand in sequence upstream of *HOM3* with *Spe*I site | This study |
| HOM3-R1 | TACTCGAGAATTCAAATTTCTTCTTGTCCGCCAC | Antisense strand in sequence downstream of *HOM3* with *Eco*RI site | This study |
| HOM3-E282D-F | TTACTCCAGAAGAAGCTTCTGATTTAACATATTATGGTTCCGAAG | Sense strand for SDM to produce *HOM3*-*E282D* | This study |
| HOM3-E282D-R | CTTCGGAACCATAATATGTTAAATCAGAAGCTTCTTCTGGAGTAA | Antisense strand for SDM to produce *HOM3*-*E282D* | This study |
| HOM3-F3 | GCAGTTGCTGTAAATGCTG | Sense strand in *HOM3* CDS | This study |
| HOM3-F4 | CATTAGTTGGTAAACATATG | Sense strand in *HOM3* CDS | This study |
| GCN4c-SphI-F | GCTTGCATGCCTGCAGGTCGACTCTAGAGG | Sense strand in vector sequences upstream of *GCN4* promoter in pYPR013 | This study |
| GCN4c-SpeI-R | ATGCACTAGTAACATGAGTACTCCTAAATAGGG | Antisense strand in vector sequences downstream of *GCN4* in pYPR013 | This study |
| GCN4-K50,K58R-F | GATTTTTGATAAATTCATCAGAACTGAAGAGGATCCAATTATCAGACAGGATACCCCTTCGAAC | Sense strand for SDM to produce *GCN4*-K50R, K58R | This study |
| GCN4-K50,K58R-R | GTTCGAAGGGGTATCCTGTCTGATAATTGGATCCTCTTCAGTTCTGATGAATTTATCAAAAATC | Antisense strand for SDM to produce *GCN4*-K50R, K58R | This study |
| GCN4-T165A-F | GACAACTTCATTCTTACCCGCTCCTGTTCTAGAAGATGC | Sense strand for SDM to produce *GCN4-T165A* | This study |
| GCN4-T165A-R | GCATCTTCTAGAACAGGAGCGGGTAAGAATGAAGTTGTC | Antisense strand for SDM to produce *GCN4-T165A* | This study |
| Primers for qRT PCR | | | |
| FZP228 | TGTGTAAAGCCGGTTTTGCC | Sense strand in *ACT1* CDS | This study |
| FZP229 | GATACCTCTCTTGGATTGAGCTTC | Antisense strand in *ACT1* CDS | This study |
| FZP237 | AGGATTTGTTAGACTATGCTGCAC | Sense strand in *ARG1* CDS | This study |
| FZP239 | GATACCTGCCTCGTAAGAAATGTG | Antisense strand in *ARG1* CDS | This study |
| FZP232 | ATGGTAAAGTTTCACCCGAAGTTG | Sense strand in *HIS4* CDS | This study |
| FZP233 | TAGTTTCTGTCCCGTAAGCCATAG | Antisense strand in *HIS4* CDS | This study |
| @75 | CGCTCCTCGTGCTGTCTTCCCATC | FAM labelled Taqman *ACT1* probe | This study |
| @497 | AGGGTATTCCCGTCGCCCAAACCA | HEX labelled *ARG1* Taqman probe | This study |
| @447+1200 | AGCAACGGCTTGGGCACCACCA | HEX labelled *HIS4* Taqman probe | This study |
| Primers for ChIP | | | |
| HQ605 | ACGGCTCTCCAGTCATTTAT | Sense strand in *ARG1* UAS sequence | [[2](#_ENREF_2)] |
| HQ607 | GCAGTCATCAATCTGATCCA | Antisense strand in *ARG1* UAS sequence | [[2](#_ENREF_2)] |
| ON265 | TAATCTGAGCAGTTGCGAGA | Sense strand in *ARG1* TATA sequence | [[3](#_ENREF_3)] |
| ON266 | ATGTTCCTTATCGCTGCACA | Antisense strand in *ARG1* TATA sequence | [[3](#_ENREF_3)] |
| HQ876' | TGGCTTATTCTGGTGGTTTAG | Sense strand in *ARG1* 5' CDS | [[3](#_ENREF_3)] |
| HQ877 | ATCCACACAAACGAACTTGCA | Antisense strand in *ARG1* 5' CDS | [[3](#_ENREF_3)] |
| HQ870 | TTCTGGGCAGATCTACAAAGA | Sense strand in *ARG1* 3' CDS | [[3](#_ENREF_3)] |
| HQ871 | AAGTCAACTCTTCACCTTTGG | Antisense strand in *ARG1* 3' CDS | [[3](#_ENREF_3)] |
| HQ1857 | CTAGAAGATTGTAGCAAGTTTG | Sense strand in chromosome V noncoding sequence | This study |
| HQ1858 | GCTTTTCCAATACGCCTTGAC | Antisense strand in chromosome V noncoding sequence | This study |

^a^CDS, coding sequence, ^b^SDM, site-directed mutagenesis

1. Winzeler EA, Shoemaker DD, Astromoff A, Liang H, Anderson K, et al. (1999) Functional Characterization of the *S. cerevisiae* Genome by Gene deletion and Parallel Analysis. Science 285: 901-906.

2. Swanson MJ, Qiu H, Sumibcay L, Krueger A, Kim S-J, et al. (2003) A Multiplicity of coactivators is required by Gcn4p at individual promoters in vivo. MolCellBiol 23: 2800-2820.

3. Qiu H, Hu C, Yoon S, Natarajan K, Swanson MJ, et al. (2004) An array of coactivators is required for optimal recruitment of TATA binding protein and RNA polymerase II by promoter-bound Gcn4p. Mol Cell Biol 24: 4104-4117.
